# Pt/Al_2_O_3_ Overcoated with Reactive Metal Oxides and Their Application to Catalytic Oxidation of Propane

**DOI:** 10.1002/smtd.202501377

**Published:** 2025-10-01

**Authors:** Geun‐Ho Han, Kunmo Koo, Selim Alayoglu, Siobhan W. Brown, Justin M. Notestein

**Affiliations:** ^1^ Department of Chemical and Biological Engineering Northwestern University 2145 Sheridan Road Evanston Illinois 60208 USA; ^2^ Center of Catalysis and Surface Science Northwestern University 2145 Sheridan Road Evanston Illinois 60208 USA; ^3^ NUANCE Northwestern University 2145 Sheridan Road Evanston Illinois 60208 USA

**Keywords:** atomic layer deposition (ALD), metal oxide catalyst, overcoat, oxidative propane dehydrogenation (ODHP), platinum catalyst, tandem catalyst

## Abstract

Inverse‐structured metal‐metal oxide materials—where the oxides are located on top of a different metal—can provide unique chemical properties. Here, a few layers of reactive metal oxides, including In_2_O_3_, MoO_3_, Bi_2_O_3_, or TiO_2_, are overcoated on Al_2_O_3_‐supported Pt nanoparticles using atomic layer deposition (ALD). In contrast to prior work focusing on stabilizing metal surfaces or new mixed‐valence nanoparticles, here the goal is to create new reactive surfaces and interfaces. The overcoating altered the Pt nanoparticle accessibility as measured by STEM, CO chemisorption, and CO DRIFTS. The reactivity of the overcoated materials is interrogated with temperature‐programmed reduction in H_2_, in propane, and in the catalytic reaction of propane with O_2_. Strong interactions between In_2_O_3_ and the Pt nanoparticles are evident from changes in Pt accessibility, In_2_O_3_ reducibility, and tandem catalytic reactivity. MoO_3_ and Bi_2_O_3_ overcoats also showed significant changes to Pt accessibility and the reducibility of the oxide in H_2_; Bi_2_O_3_ addition led to complete propane combustion. This study establishes ALD methods for reactive oxides on high surface area materials suitable for applications such as heterogeneous catalysis, and it illustrates the wide range of useful physiochemical modifications resulting from the unique oxide‐metal interfaces generated.

## Graphical abstract

Inverse structured nanomaterials are synthesized by atomic layer deposition of reactive metal oxides (In_2_O_3_, MoO_3_, Bi_2_O_3_, or TiO_2_) on top of Al_2_O_3_‐supported Pt nanoparticles. The morphology, stability, and reactivity of the nanometer‐thin oxide layers are altered by the underlying Pt nanoparticles, with specific improvements to oxide reducibility and hydrocarbon activation.

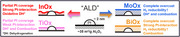

## 1 Introduction

**Scheme 1:**
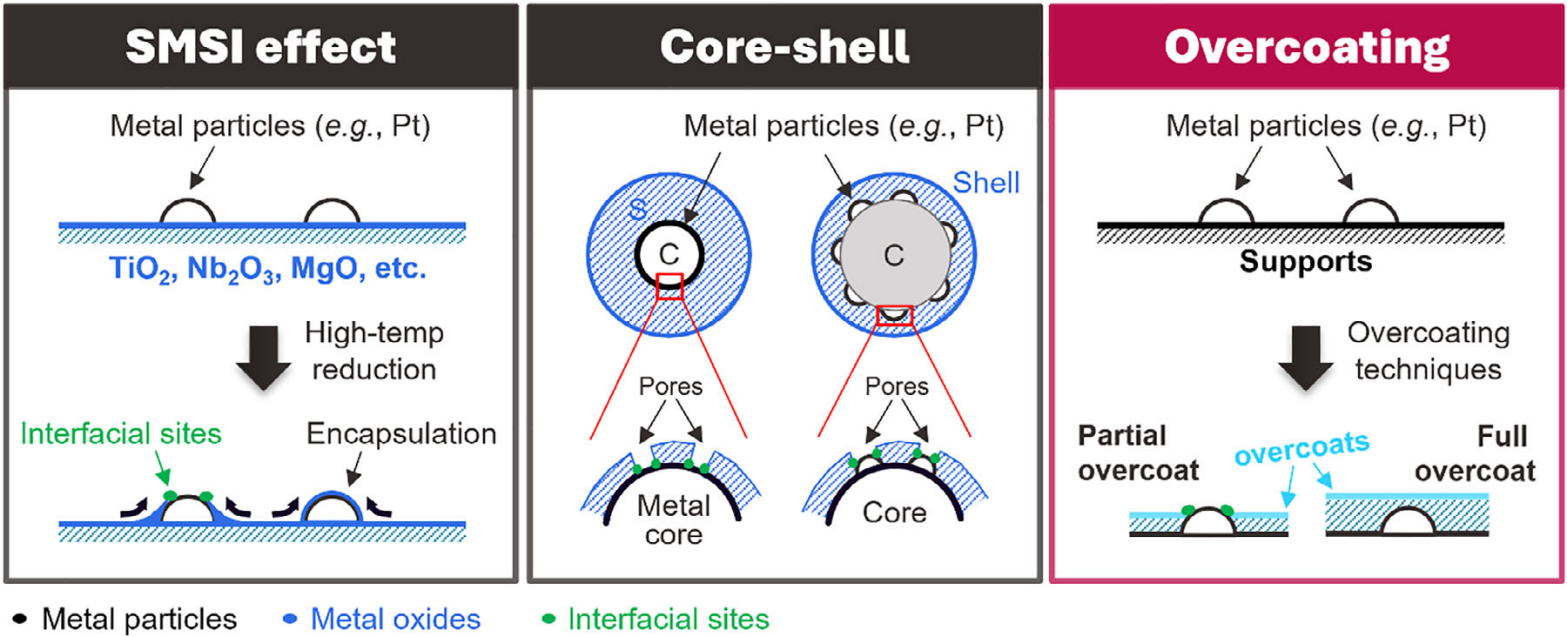
Types of overcoated catalytic materials. (Left) Spontaneously‐formed, strong metal‐support interaction (SMSI) structures, (Center) core‐shell structures having a metal core and a metal oxide shell, and (Right) overcoated supported metal nanoparticles by deliberate synthesis.

In heterogeneous catalysis, metal oxides are frequently supports that immobilize metal nanoparticle catalysts from “underneath”. Far from inert, they can modulate the electronic states of active metals or form novel sites at the metal‐support interface.^[^ ^1^, ^2^ ^]^ In this light, inverse structures – where metal oxides are overcoated on top of supported metals – can provide unique and unprecedented properties. When it occurs spontaneously, this phenomenon is known as the Strong Metal‐Support Interaction (SMSI) effect, in which supports such as TiO_2_, Nb_2_O_5_, or MgO climb up and over supported metal nanoparticles to form thin sub‐oxide or carbonate layers under reducing conditions (**Scheme** **1**, left).^[^ ^3^, ^4^, ^5^ ^]^ Such inverse structures can also be synthesized deliberately as core‐shell nanoparticles consisting of core metal nanoparticles with oxide overlayers (Scheme 1, Center).^[^ ^5^ ^]^ We are very interested in designing and synthesizing overcoated, supported metal nanoparticles (inverse structures), akin to an artificial SMSI, but with a broader compositional range.

As a synthesis tool, the SMSI effect is limited because it is a spontaneous process that requires specific compositions (e.g., reducible bulk oxides like TiO_2_ as supports) and reducing conditions.^[^ ^6^, ^7^, ^8^, ^9^ ^]^ Conversely, there is a very large synthesis literature base for core‐shell particles, but the majority of these materials utilize relatively large nanoparticles, thick shells, and are aimed primarily at *protecting* the metal nanoparticles, rather than creating new properties for the metal and the oxide.^[^ ^10^, ^11^ ^]^ Moreover, the recipe‐driven sol‐gel methods often employed to synthesize these materials have extensive liquid handling and multiple washing/collection steps that can lead to reproducibility challenges, unintended surface modification, and solids loss.^[^ ^12^, ^13^, ^14^ ^]^ Therefore, direct overcoating strategies that deposit only a few layers of oxide are expected to provide more control and increased compositional/structural diversity to inverse oxide materials. Importantly, direct overcoating strategies will allow modification of the huge diversity of existing, supported metal catalysts (Scheme 1, Right).^[^ ^15^ ^]^

One route to direct overcoating is through atomic layer deposition (ALD). ALD is a self‐limiting, highly conformal, thin film deposition technique that was originally developed for semiconductor manufacturing and further applied to batteries, sensors, transistors, and solar cells.^[^ ^15^, ^16^, ^17^, ^18^, ^19^, ^20^, ^21^, ^22^, ^23^, ^24^, ^25^, ^26^, ^27^, ^28^, ^29^ ^]^ ALD achieves self‐limiting deposition due to specific chemical reactions between ALD precursors (e.g., trimethylaluminum) and functional groups (e.g., hydroxyl groups) on material surfaces, leading to Angstrom or monolayer‐level thickness control.^[^ ^30^ ^]^ This conformal and precise deposition is particularly useful for high‐aspect structures such as heterogeneous catalysts with pores,^[^ ^15^, ^16^ ^]^ which motivates some of the development of new ALD methods and applications.^[^ ^17^, ^31^, ^32^, ^33^, ^34^, ^35^ ^]^ In addition, most elements can be deposited, and not only as oxides.^[^ ^15^ ^]^ Compared to other wet chemistry routes like colloidal nano‐synthesis, ALD does not necessarily require further treatments to remove organics (e.g., polymers or surfactants), and as‐synthesized materials can be applied directly.

The ALD technique has been used to significant effect in overcoated catalysts.^[^ ^36^, ^37^, ^38^, ^39^ ^]^ For example, Lu et al. achieved a coking‐ and sintering‐resistant Pd catalyst by using an Al_2_O_3_ overcoat.^[^ ^36^ ^]^ They found that relatively thick Al_2_O_3_ overcoats formed a few nanometers of microporous overlayer, which prevented heavy coke accumulation and sintering of Pd particles.^[^ ^36^ ^]^ Canlas et al. utilized Al_2_O_3_ overlayers with sacrificial organic templates as shape‐selective sieves.^[^ ^39^ ^]^ The space occupied by the templates formed cavity structures above the active species, which selected reactant molecules based on size.^[^ ^39^ ^]^ Ni with an Al_2_O_3_ overcoat increased and stabilized the number of Ni‐Al_2_O_3_ interfacial sites responsible for cinnamaldehyde and nitrobenzene hydrogenation.^[^ ^40^ ^]^ Similarly, TiO_2_ and Al_2_O_3_ overcoats changed the kinetics of CO desorption from Pt by creating new interfacial sites.^[^ ^41^ ^]^

Most studies of these inverse or overcoated materials address the reactivity of the metal species, including the formation of interfacial sites, charge transfer, and steric hindrance at the metal surface.^[^ ^40^, ^42^, ^43^ ^]^ Likewise, most oxide overcoats employed, such as SiO_2_ or Al_2_O_3_, are relatively inert under reaction conditions. Here, we focus on reactive metal oxides that might actively participate in bond breaking and bond making, and we seek to understand how reactivity differs between a conventional oxide support and when the same oxide is used as an overcoat over Pt nanoparticles. In the limit, some systems of overcoated metal nanoparticles may show novel reactivity emerging from the metal and oxide working in tandem.

Relatively few studies have examined how proximate metal nanoparticles change the reactivity of an adjacent oxide film or nanoparticle. Spillover of H atoms from metals to oxides is one well‐documented example, and spillover to oxides like MoO_3_ can generate new acid sites.^[^ ^44^, ^45^ ^]^ In a more physical change, metal nanoparticles can decrease the glass transition temperature of amorphous SiO_2_, leading to nanoparticle entrenching behavior.^[^ ^46^, ^47^, ^48^ ^]^ Stone et al. reported that a core‐shell Pt@Al_2_O_3_‐CeO_2_ was more reducible than the Pt‐free Al_2_O_3_‐CeO_2_.^[^ ^49^ ^]^ Christopher et al. demonstrated that the restructuring of TiO_2_ during CO_2_ reduction created persistent, carbonate‐containing overlayers on Rh nanoparticles that displayed reactivity distinct from that of any of the starting materials.^[^ ^3^ ^]^ As the metal‐oxide connection grows tighter, the two sites can work together as tandem catalysts that can couple mechanistically distinct reactions into a single reaction system.^[^ ^2^, ^50^, ^51^, ^52^, ^53^ ^]^ These are much desired to improve the energy‐ and atom‐efficiency of chemical processing.^[^ ^37^, ^54^ ^]^ Some of us have shown that a Pt nanoparticle catalyst with an ALD‐derived In_2_O_3_ overcoat carries out propane dehydrogenation in tandem with selective hydrogen combustion on Pt and In_2_O_3_, respectively, for enhanced propylene yields.^[^ ^37^ ^]^ Many bifunctional catalysts, including ones with an overcoat geometry, have been developed that combine metal and acid functionality for dehydration + hydrogenation,^[^ ^55^ ^]^ Fischer‐Tropsch + cracking,^[^ ^56^ ^]^ or CO_2_ hydrogenation + coupling.^[^ ^57^, ^58^, ^59^ ^]^ These latter examples may benefit from the proximity of two types of sites that communicate via fluid‐phase intermediates, but they do not necessarily require direct transfer of surface species from one active site to another.

Here, we will build off reports of ALD of oxides like V_2_O_5_ and MoO_3_^[^ ^60^, ^61^ ^]^ that are active for H─H and/or C─H bond cleavage and investigate how the structure and reactivity of the oxide change when overcoated over Al_2_O_3_‐supported Pt nanoparticles (Pt/Al_2_O_3_). We use In_2_O_3_, MoO_3_, Bi_2_O_3_, and TiO_2_. Some of us and others have reported ALD modification of Pt catalysts with TiO_2_ and In_2_O_3_.^[^ ^37^, ^62^, ^63^, ^64^, ^65^ ^]^ In_2_O_3_ is well‐known for CO_2_ hydrogenation and selective hydrogen combustion, and is of extensive current interest.^[^ ^37^, ^66^, ^67^, ^68^, ^69^, ^70^, ^71^, ^72^ ^]^ TiO_2_ overcoats form spontaneously under reducing conditions with TiO_2_‐supported metal nanoparticles; these are the canonical SMSI materials.^[^ ^73^, ^74^, ^75^, ^76^ ^]^ Conversely, we believe this is the first report of ALD‐derived MoO_x_ or BiO_x_ modifying a Pt catalyst. Supported MoO_x_ is known for H_2_ activation and alkane dehydrogenation, among many other reactions.^[^ ^45^, ^77^, ^78^, ^79^ ^]^ BiO_x_ is a common promoter or active component in the dehydrogenation and selective oxidation of alkanes and H_2_.^[^ ^78^, ^80^, ^81^, ^82^ ^]^ Combinations of these latter two oxides with Pt are especially important in industrial chemistry.^[^ ^83^, ^84^, ^85^, ^86^, ^87^ ^]^

We study the physical and chemical characteristics of these ALD‐derived In_2_O_3_‐, MoO_3_‐, Bi_2_O_3_‐, or TiO_2_‐modified Pt/Al_2_O_3_ materials. The overall structure is examined by N_2_ physisorption, X‐ray diffraction (XRD), and Scanning Transmission Electron Microscopy (STEM). The electronic states of the overcoats and Pt are investigated by X‐ray photoelectron spectroscopy (XPS). Pt accessibility is interrogated with CO chemisorption, CO Diffuse Reflectance Infrared Fourier Transform Spectroscopy (DRIFTS), and STEM. The reactivity of the oxides and the composite materials is seen through temperature programmed reaction with H_2_ and propane, and we look for changes in reactivity in catalytic propane oxidation. In the latter case, we are interested in cases that either give improved selectivity toward propylene by coupling propane dehydrogenation with selective H_2_ combustion,^[^ ^16^, ^88^, ^89^, ^90^, ^91^, ^92^, ^93^, ^94^ ^]^ or systems that show improved total combustion reactivity. Here, MoO_3_ and Bi_2_O_3_ show good evidence of chemical and physical interaction between the metal and oxide components, and Bi_2_O_3_ improves the total combustion. In_2_O_3_ remains unique in its ability to promote the tandem reaction between propane dehydrogenation and selective H_2_ combustion.

## 2 Experimental Methods

Experimental methods are described in full in the Supporting Information, but the procedures are summarized here. The base Pt/Al_2_O_3_ catalyst was prepared by wet impregnation of Al_2_O_3_ (NanoArc, Alfa Aesar) with tetraamineplatinum(II) nitrate ((NH_3_)_4_Pt(NO_3_)_2_, Thermo Scientific) at a target loading of ≈1 wt.% Pt. Residual H_2_O was evaporated in a rotary evaporator at 50 °C under dynamic vacuum overnight, then the powder was sieved and calcined at 300 °C for 4 h in static air. In_2_O_3_, MoO_3_, Bi_2_O_3_, and TiO_2_ were added using ALD, with parameters summarized in **Table** **1** and procedures described in detail in the Supporting Information.

**Table 1:** Major controllable parameters for In, Mo, Bi, and Ti deposition by ALD synthesis over Al_2_O_3_ and Pt/Al_2_O_3_ materials, contents of overcoating materials, and CO pulse chemisorption data under different pretreatments.

Overcoated materials	ALD recipe								Overcoat content [wt.%]^c)^	Loss of Pt surface [%]^d)^ (Fresh / 450 °C He pretreated)
	Temperatures (°C)^a)^			Cycle A		Cycle B		Total cycles		
	Bottle	Manifold	Chamber	Metal ^b)^	Exposure time [s]	Osource	Exposure time [s]			
In_2_O_3_@Al_2_O_3_	50	115	150	InCp	450	O_3_+H_2_O	200	8	7.3	–
In_2_O_3_@(Pt/Al_2_O_3_)									6.6	85 / 77
MoO_3_@Al_2_O_3_	50	115	150	Mo(CO)_6_	24	O_2_ plasma	50	20	6.2	–
MoO_3_@(Pt/Al_2_O_3_)									5.7	100 / 75
Bi_2_O_3_@Al_2_O_3_	140	150	250	Bi(Ph)_3_	30	O_3_	200	10	13.1	–
Bi_2_O_3_@(Pt/Al_2_O_3_)									13.3	100 / 100
TiO_2_@Al_2_O_3_	80	115	200	TTIP	5	H_2_O	8	100	2.9	–
TiO_2_@(Pt/Al_2_O_3_)									3.1	74 / 40

^a)^ Three key parts of the ALD instrument. See Scheme S1 (Supporting Information);

^b)^ ALD metal precursors. InCp; cyclopentadienylindium(I), Mo(CO)_6_; molybdenum hexacarbonyl, Bi(Ph)_3_; triphenylbismuth, and TTIP; titanium(IV) iso‐propoxide;

^c)^ Of In, Mo, Bi, or Ti, as measured by ICP‐OES;

^d)^ Calculated by (1 – CO_overcoated_ / CO_Pt/Al2O3_) 𝖷 100. A value of 100 indicates that Pt particles are fully covered without measurable CO adsorption sites remaining.

N_2_ adsorption‐desorption isotherms were obtained at 77K using a Micromeritics 3FLEX instrument in the REACT core facility at Northwestern University after degassing at 150 °C overnight. Specific surface area (S_BET_) and pore properties were derived from the Brunauer–Emmett–Teller (BET) equation and the Non‐Local Density Functional Theory (NLDFT) model, respectively. Transmission electron microscopy (TEM) and scanning transmission electron microscopy (STEM) images were taken in the NUANCE facility at Northwestern University using a JEOL ARM200CF probe‐corrected field‐emission transmission electron microscope with an acceleration voltage of 200 keV, probe diameter of 1 Å, and 62 pA probe current at the STEM imaging condition. Materials were dispersed in methanol and drop‐cast on lacey carbon (Ted Pella). X‐ray photoelectron spectra (XPS) were collected by using a NEXSA G2 (Thermo Scientific, Al Kɑ radiation, 1486 eV) with an electron flood gun in the NUANCE facility at Northwestern University. Elemental analysis was carried out using ICP‐OES (Thermo iCap7600 ICP‐OES) of acid‐digested samples in the QBIC center at Northwestern University. Please see the Supporting Information for detailed sample digestion and analysis procedures. Powder X‐ray diffraction (PXRD) patterns were collected on a STOE‐STADI P diffractometer using CuKα1 radiation.

Temperature‐programmed reduction by hydrogen (H_2_‐TPR) or propane (C_3_‐TPR) was performed using an AutoChem II 2920 (Micromeritics) with onboard TCD coupled to an online mass spectrometer (SRS Universal gas analyzer, UGA) in the REACT core facility at Northwestern University. Materials were packed into a U‐type quartz tube with quartz wool and silicon carbide used to maintain the height and volume of the material bed in the tube. Samples were pretreated at 200 or 450 °C under 20 sccm He for 1 h. After cooling and stabilization, TPR was carried out with a ramp to 550 at 10 °C min^−1^ under 40 sccm of 4% H_2_/Ar or 10% propane/Ar. Sensitivity factors were used to calculate the molar flow of each effluent gas. Degree of reduction (DOR) was defined as the moles of H_2_O evolved until 450 °C, divided by the total number of metal atoms in the overcoat (In, Mo, Bi, or Ti) from ICP‐OES analysis. DOR is expressed as an absolute value and is not normalized for the stoichiometry of the bulk oxide. Carbon monoxide (CO) pulse chemisorption was performed using the same sample loading procedures and pretreatment as above. A 1:1 CO:Pt_surf_ adsorption stoichiometry was assumed.

CO probe, diffuse reflectance infrared Fourier transform spectroscopy (DRIFTS) was used a Thermo Nicolet 6700 FTIR spectrometer equipped with a praying mantis diffuse reflectance sample holder in the REACT core facility at Northwestern University. Materials were loaded inside the sample holder without dilution. Samples were pretreated at 200 °C under 100 sccm Ar for 1 h, cooled to 40 °C, and backgrounds were acquired. 100 sccm of 5% CO/Ar was fed until the gas phase CO vibrational bands were saturated. Then, the holder was flushed with 100 sccm Ar, and the experimental spectra were acquired (64 scans, 4 cm^−1^ resolution, Kubelka – Munk transform for data presentation).

Catalytic propane oxidation was carried out in a U‐type quartz tube reactor with 100 mg of catalyst diluted 1:4 with quartz sand. Void volume after the catalyst bed was filled with quartz sand to suppress gas‐phase reactions. Catalysts were pretreated at 450 °C under He for 1 h, followed by 6 h under a reaction mixture of 5 sccm 20% propane/He and 5 sccm 10% O_2_/He, giving a gas‐hourly space velocity (GHSV) of 600 sccm_C3_ g_cat_^−1^ h^−1^. Product analysis was via online gas chromatograph (Shimadzu, GC‐2014) with a Carboxen 1010 PLOT (30 m x 0.53 mm ID) connected to a TCD for O_2_, CO, and CO_2_, and a GS‐Gaspro (30 m x 0.32 mm ID) connected to an FID for hydrocarbon analysis.

## 3 Results and Discussion

### 3.1 Catalytic Reaction of the Metal Oxide‐Overcoated Al_2_O_3_ and Pt/Al_2_O_3_

**Scheme 2:**
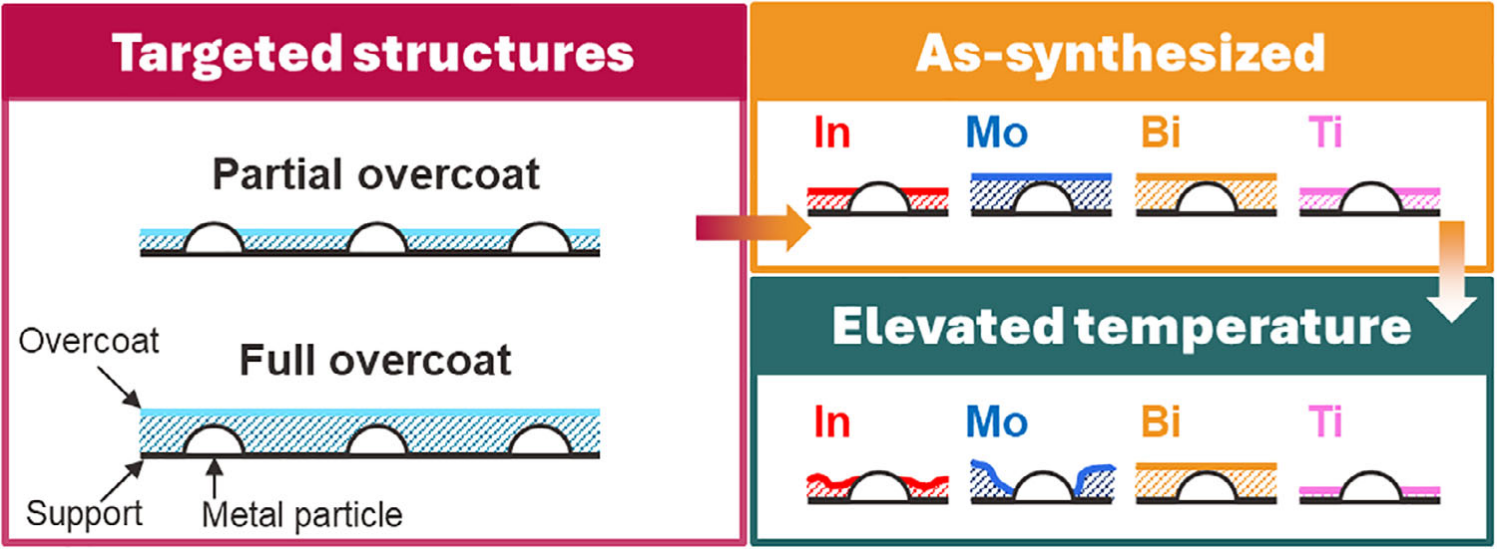
Illustration of the targeted structures of overcoated materials, the as‐synthesized structures, and the structures that result after treatment at elevated temperatures.

Pt/Al_2_O_3_ powder was prepared by wet impregnation at 0.8 wt.%, as confirmed by ICP‐OES. In Figure S1a (Supporting Information), Pt/Al_2_O_3_ shows the mixed X‐ray diffraction pattern of gamma and delta alumina of the support. Pt is not observed due to the low Pt content (0.8 wt.%) and/or small particle size.^[^ ^95^ ^]^ In Figure S1b (Supporting Information), the N_2_ adsorption‐desorption isotherms of Pt/Al_2_O_3_ and Al_2_O_3_ show typical type H3 isotherms and surface areas of ≈40 m^2^ g^−1^ (Table S1, Supporting Information). In Figure S1c (Supporting Information), STEM of fresh Pt/Al_2_O_3_ shows small Pt particles with an average size of 1.2 nm. CO pulse chemisorption gives a larger average Pt particle size of 2.9 nm, which includes the effect of the minority of larger Pt particles. Hereafter, Pt/Al_2_O_3_ and Al_2_O_3_ serve as substrates to deposit reactive metal oxides (i.e., In_2_O_3_, MoO_3_, Bi_2_O_3_, and TiO_2_) on top of them. **Scheme** **2** illustrates the targeted and resulting oxide overcoats on the powder Pt/Al_2_O_3_ materials. In the following pages, we will demonstrate that the as‐synthesized structures fall between partial and full coverage of the Pt nanoparticles. We will also discuss how the structures evolve at high temperatures representative of industrial catalytic reactions.

**Figure 1:**
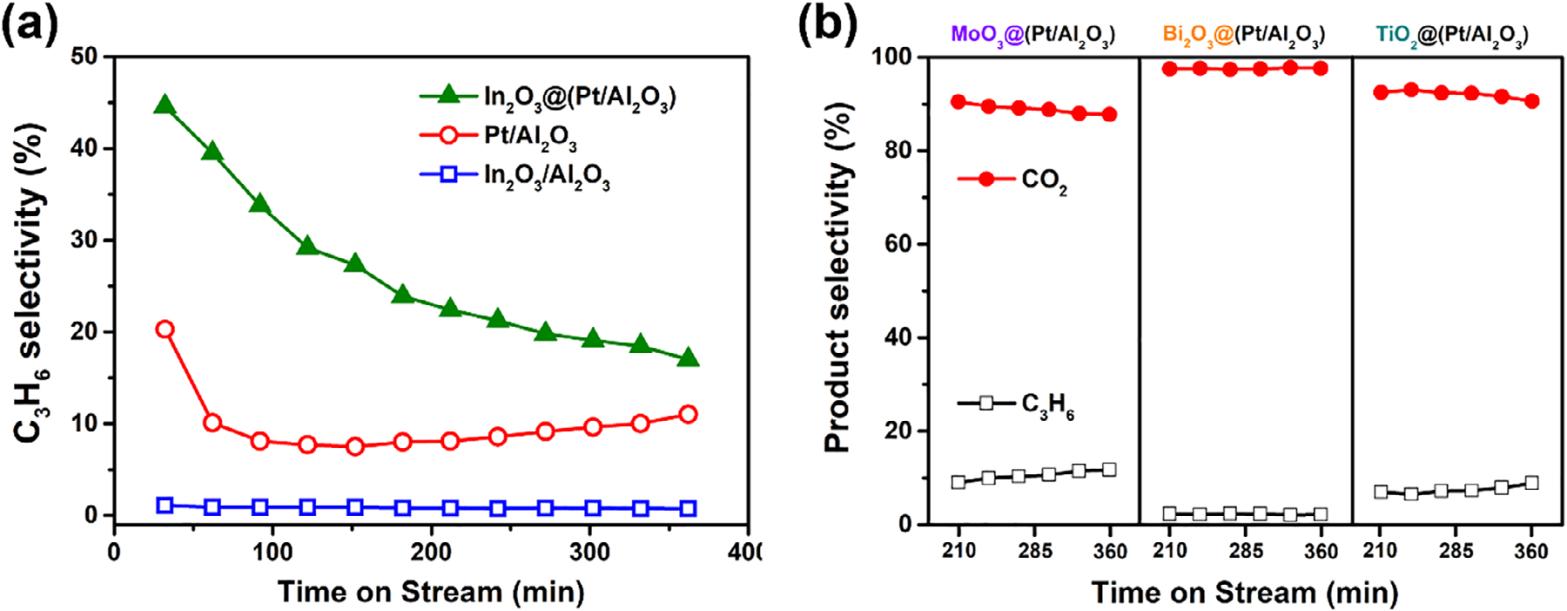
a) Propylene selectivity of Pt/Al_2_O_3_, In_2_O_3_/Al_2_O_3_, and In_2_O_3_@(Pt/Al_2_O_3_). Improved selectivity is evidence of tandem (PDH+SHC) reactivity. b) Product selectivity (● CO_2_, □ propylene) of MoO_3_@(Pt/Al_2_O_3_), Bi_2_O_3_@(Pt/Al_2_O_3_), and TiO_2_@(Pt/Al_2_O_3_). Bi_2_O_3_@(Pt/Al_2_O_3_) is unusually selective to CO_2_ even at ≈100% O_2_ conversion. Conditions: 450 °C 1 h under He, then 600 sccm_C3_ g_cat_^−1^ h^−1^ GHSV in 10% propane, 5% O_2_, balance He.

To motivate the characterization of these materials, we first summarize the performance of the overcoated nanomaterials as catalysts for the reaction of 2:1 propane: O_2_ (**Figure** **1**). Additional details are given in Figure S2 (Supporting Information). These conditions are stoichiometric for the oxidative dehydrogenation to propylene but will lead to combustion over many catalysts, which provides insights into the physicochemical and reactive properties of the synthesized materials. Note that 10% is the maximum conversion under these conditions if combustion is the exclusive reaction. Figure 1a shows that In_2_O_3_ is exclusively a combustion catalyst, while Pt/Al_2_O_3_ gives minor selectivity to propylene and high O_2_ conversion (see Figure S2a, Supporting Information), consistent with serial total combustion, followed by propane dehydrogenation after all O_2_ is consumed. Conversely, the In_2_O_3_‐overcoated Pt/Al_2_O_3_ demonstrates significantly higher propylene selectivity and reduced combustion. Multiple batches of the In_2_O_3_@(Pt/Al_2_O_3_) were synthesized and tested, and their reactivity matches that of the first batch, demonstrating the reproducibility of this method (Figure S3, Supporting Information). Some of us have previously proposed that the synergistic effect of In_2_O_3_‐overcoated Pt/Al_2_O_3_ is the result of a tandem catalytic network ^[^ ^37^ ^]^ instead of the formation of more sites that are specifically selective for non‐oxidative propane dehydrogenation (Figure S4, Supporting Information). In this tandem catalyst network, partially exposed Pt sites dehydrogenate propane to propylene and H atoms, and the H atoms are rapidly consumed near the Pt‐In_2_O_3_ interface. Rapid, selective H_2_ consumption on or near In_2_O_3_ provides local heating and pulls forward the endothermic propane dehydrogenation reaction, while also clearing the surface of O_2_ to decrease propane combustion on Pt.

Conversely, MoO_3_ and TiO_2_‐overcoated Pt/Al_2_O_3_ exhibit nearly the same reactivity as Pt/Al_2_O_3_ (Figure 1b), indicating that the oxide‐metal interface does not generate new sites for propane or O_2_ activation that can outcompete those that already exist on Pt. Finally, the Bi_2_O_3_‐overcoated catalyst almost exclusively carries out combustion, even under these fuel‐rich conditions and complete O_2_ consumption (see Figure S2g, Supporting Information), suggesting the formation of new sites that are very effective for combustion and that the dehydrogenation reactivity of Pt is suppressed. To understand the origin of these three distinct behaviors (enhanced selectivity to dehydrogenation, enhanced selectivity to combustion, and no kinetically relevant effect), we further investigated the physicochemical properties of the overcoated materials. Surface morphology, interaction between Pt and overcoats, reducibility, and stability all can influence their reactivity and are addressed in the pages that follow.

### 3.2 Physicochemical Properties of the Metal Oxide‐Overcoated Al_2_O_3_ and Pt/Al_2_O_3_

Table 1 is a summary of ALD method development, including the major controllable parameters. Bottle/manifold temperatures were chosen to increase metal precursor partial pressures, and chamber temperatures were chosen to enable the surface reactions without self‐decomposition within the “ALD window” for each precursor.^[^ ^15^ ^]^ Total oxide deposition was controlled by the total cycles alternating precursor (A) and O source (B). The number of cycles required for a given level of deposition varied substantially from precursor to precursor. Details on method development are found in the Section S1.2, Supporting Information, and are adopted from references on ALD of the various oxides in the absence of any metal nanoparticles.^[^ ^42^, ^96^, ^97^, ^98^, ^99^, ^100^ ^]^

In this study, we targeted a deposition of ≈10 atoms of metal (In, Mo, Bi, or Ti) per nm^2^ of the parent Al_2_O_3_ support, or 1–3 monolayers depending on the density of the deposited oxide. We had the goal of forming a complete film of the oxide, avoiding both separated islands of metal oxides and excessively thick films. Similar atomic densities result in varied contents (wt.%) based on the atomic mass of overcoating metals (Table 1). In the first step of ALD, the metal precursors react with hydroxyl groups on Al_2_O_3_.^[^ ^30^ ^]^ Precursors such as TTIP or InCp undergo ligand exchange with the surface hydroxyls to directly form (metal)─O─Al bonds and liberate an isopropoxy or cyclopentadienyl group.^[^ ^96^, ^101^, ^102^ ^]^ Similarly, grafting of Mo(CO)_6_ is expected to yield sub‐carbonyls during the grafting step.^[^ ^103^ ^]^ The ligands of the Bi(Ph)_3_ precursor are stable after the initial grafting.^[^ ^98^ ^]^ In the second step, any remaining ligands are removed by reaction with O sources, also generating additional (metal)─O─Al bonds. TTIP requires only H_2_O, as the Ti atom is already in its highest oxidation state, and the isopropoxy groups are readily hydrolyzed. For the Mo, In, and Bi precursors, O_2_ or O_3_ is required to remove stable ligands, oxidize the metal center, or both.

When using the same ALD method, there is less In_2_O_3_ or MoO_3_ deposited on Pt/Al_2_O_3_ than is deposited on Al_2_O_3_. This suggests that the deposition of these two species is primarily on the Al_2_O_3_ surface, and that the Pt NP physically blocks some available surface on which the oxide could grow. Conversely, Bi_2_O_3_ and TiO_2_ grow marginally faster on Pt/Al_2_O_3_ than on Al_2_O_3_, suggesting that Pt may catalyze precursor deposition or help activate the oxidant on these materials. This hypothesis is supported by literature reports of Pt, Ru, or Ni catalyzing the deposition of Pd(hfac)_2_ at relatively mild ALD conditions.^[^ ^104^, ^105^ ^]^ Likewise, vanadium oxide layers have been shown to facilitate bismuth oxide deposition.^[^ ^106^ ^]^ We next interrogate the physical and chemical properties of the overcoated materials to assess where the precursors are deposited and how they interact with the Pt NPs.

In Table S1 (Supporting Information), specific surface areas (S_BET_) of the overcoated nanomaterials derived from N_2_ physisorption isotherms (Figure S5a, Supporting Information) are only marginally lower than those of the parent materials (i.e., Al_2_O_3_ or Pt/Al_2_O_3_), and the shape of the isotherms is unchanged. This small decrease in S_BET_ is consistent with additional deposition of the heavier atoms in the overcoats and/or minor porosity blocking by the overcoats. Overcoating does not result in new, distinct crystalline features (Figure S5b, Supporting Information), and the absence of new features in XRD indicates that the deposited oxide overcoats are amorphous and/or possess very small crystalline domains.

XPS spectra show that the ALD oxides are in their highest oxidation states (In^3+^, Mo^6+^, Bi^3+^, and Ti^4+^, solid lines in Figure S6a,b,d, and e, Supporting Information^[^ ^107^, ^108^, ^109^, ^110^, ^111^, ^112^, ^113^, ^114^ ^]^) whether deposited on Al_2_O_3_ or on Pt/Al_2_O_3_. We did not expect Pt to induce changes in the average properties of the ALD overcoats because the majority of the overcoats are on bare Al_2_O_3_ and not in proximity to Pt nanoparticles. Conversely, the use of small Pt nanoparticles ensures that most Pt atoms can be in proximity to an overcoat, and the electronic state of Pt is indeed sensitive to the addition of the oxide overcoats (Figure S7a, Supporting Information), immediately after synthesis. In_2_O_3_ and TiO_2_ overcoats shifted the Pt peak to lower energies, indicating more electron‐rich Pt than for bare Pt/Al_2_O_3_. Conversely, MoO_3_ overcoats shifted the Pt peak to higher energies, indicating an electron‐deficient Pt. Bi_2_O_3_ overcoats do not notably change the Pt spectrum. After reaction in propane/O_2_, all spectra were indistinguishable from that of Pt/Al_2_O_3_ (Figure S7b, Supporting Information), indicating that any changes in catalytic reactivity after overcoating are more likely due to the presence of the nearby oxide, rather than to very minor changes to the electronic state of the Pt nanoparticles themselves.

**Figure 2:**
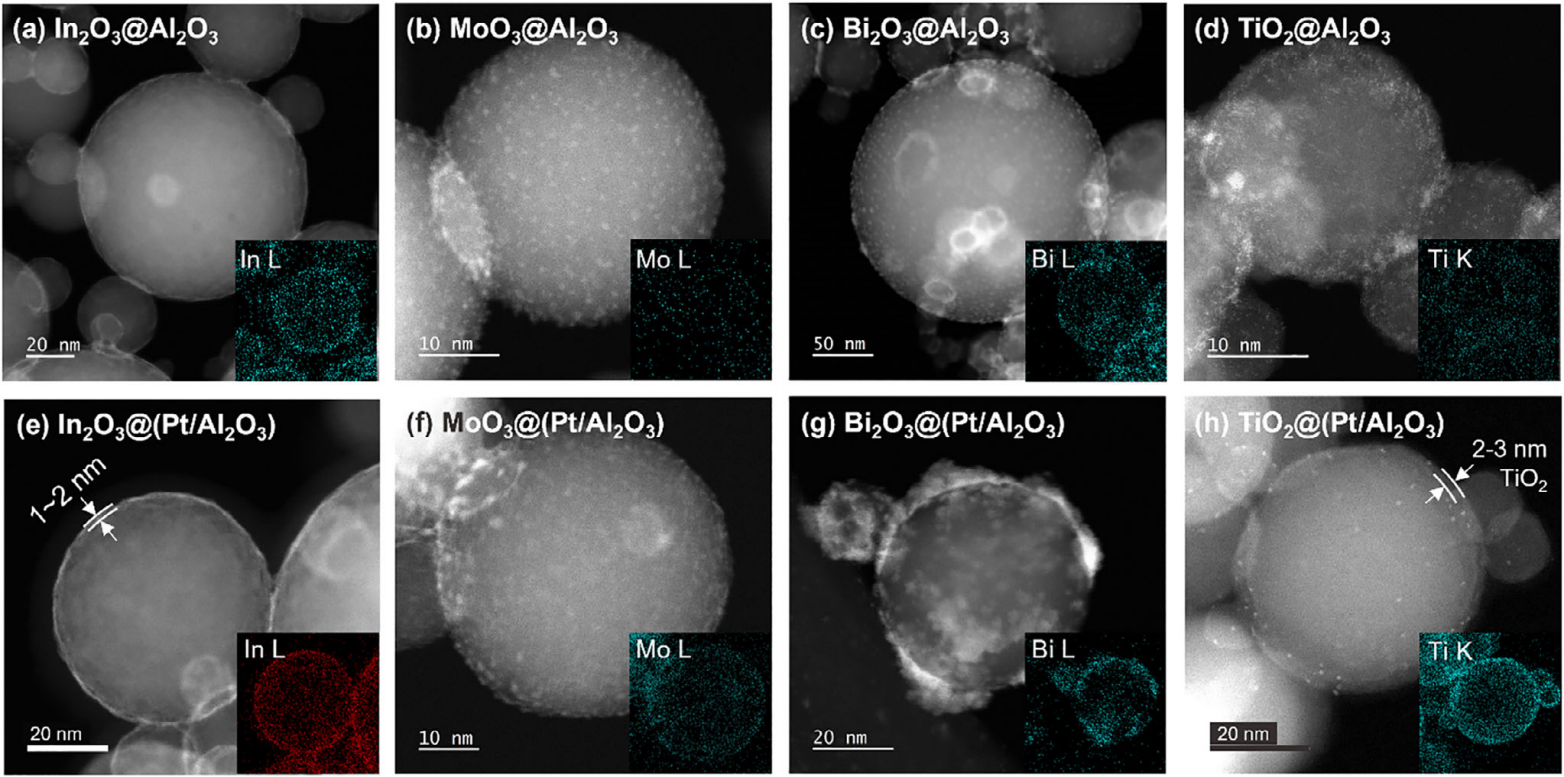
STEM images of as‐synthesized, ALD‐overcoated nanomaterials with inset EDS maps for overcoating elements (In, Mo, Bi, and Ti). (top row) MOx@Al_2_O_3,_ where M is In a), Mo b), Bi c), and Ti d), and (bottom row) MOx@(Pt/Al_2_O_3_) where M is In e), Mo f), Bi g), and Ti h).

Next, all materials are imaged by STEM to observe the morphology of nanomaterials. **Figure** **2**a,e shows conformal In_2_O_3_ layers on Al_2_O_3_ and Pt/Al_2_O_3_. These materials possess a 1.3–1.4 nm bright layer (Figure S8c,d, Supporting Information) not found on pristine Al_2_O_3_ (Figure S1c, Supporting Information), which we attribute to the In atoms. The inset EDS map shows the In signal localized along the boundary of the particle, indicating conformal deposition of In_2_O_3_. For MoO_3_ and Bi_2_O_3_ overcoats, (Figure 2b,c; Figure S8a,b,e, and g, Supporting Information) we observe 0.8 and 2.3 nm clusters on Al_2_O_3_. The MoO_3_ overcoat has a similar morphology on Al_2_O_3_ or Pt/Al_2_O_3_ (Figure 2f and Figure S7f, Supporting Information), whereas the Bi_2_O_3_ overcoat is uneven and flocculent on Pt/Al_2_O_3_ (Figure 2 g and Figure S7h, Supporting Information). Finally, TiO_2_ was deposited as very small clusters on bare Al_2_O_3_ (Figure 2d) but formed a 3.3 nm layer of conformal TiO_2_ on Pt/Al_2_O_3_ (Figure 2 h and Figure S8i, Supporting Information). The ≈2 nm Pt particles can be seen embedded in the TiO_2_ layer. From these observations, all oxides substantially cover the underlying powders, but the structures of the Bi_2_O_3_ and the TiO_2_ layers are altered by the presence of the Pt nanoparticles. This change in the overcoat morphology is consistent with the change in the deposited amounts given in Table 1 and again implies that the Pt nanoparticles are locally catalyzing the ALD reactions. Conversely, the growth of MoO_3_ and In_2_O_3_ appears to be generally unaffected by the presence of the Pt nanoparticles.

Although XPS shows that overlayer deposition does not significantly change the electronic structure of the Pt nanoparticles, especially after oxidative reactions, the ALD may physically block the surface and decrease the absolute number of sites available to bind reactants or change the relative amounts of the different exposed Pt sites (e.g., terraces vs edge and corner sites). Therefore, possible physical blocking of the Pt NPs was first investigated by CO pulse chemisorption. Table 1 reports the percentage loss of accessible Pt surface after overcoating by the four oxides. Immediately after deposition, the In_2_O_3_ and TiO_2_ overcoats blocked 85 and 74% of the Pt NP surface, respectively. As seen in STEM, the overcoats formed conformal films across the Al_2_O_3_ powder, but the CO chemisorption results show that some of the Pt nanoparticles remain partially exposed. Conversely, MoO_3_ and Bi_2_O_3_ overcoats resulted in a 100% loss of accessible Pt surfaces. Even though these oxides are deposited as visible clusters rather than even films, there must be preferential deposition on/near the Pt NPs. Overall, this characterization leads to the representative illustrations in Scheme 2b.

Since ALD synthesis is often conducted at relatively mild conditions compared to many industrially‐relevant catalytic reactions, elevated temperatures can restructure the as‐synthesized materials to have different physicochemical properties.^[^ ^37^, ^115^ ^]^ Therefore, we re‐assessed Pt surface blocking after heat treatment at 450 °C in inert (He) (Table 1). After heat treatment, marginally less of the Pt NP surface in In_2_O_3_@(Pt/Al_2_O_3_) was blocked (85% to 77% coverage), indicating a stable interface between Pt and the overcoat. Bi_2_O_3_ continued to completely encapsulate the Pt NPs even after the heat treatment. This demonstrates a strong and stable interaction between Pt and Bi_2_O_3_ across the entire surface, which is likely responsible for the near‐total suppression of dehydrogenation reactivity for this material (Figure 1b, Figure S2f,g, Supporting Information). After heat treatment of MoO_3_@(Pt/Al_2_O_3_), the Pt NP surface was partially uncovered, moving from 100% coverage to 75% coverage. Finally, for TiO_2_@(Pt/Al_2_O_3_), the Pt NP coverage decreased drastically from 74% to 40% after heat treatment. Overall, these changes to CO chemisorption demonstrate that the Pt‐In_2_O_3_ and Pt‐Bi_2_O_3_ interfaces are relatively stable, while the Pt‐MoO_3_ and Pt‐TiO_2_ interfaces are noticeably changed by elevated temperature treatments. After these treatments, In_2_O_3_@(Pt/Al_2_O_3_), MoO_3_@(Pt/Al_2_O_3_), and TiO_2_@(Pt/Al_2_O_3_) expose both oxide and metal surfaces, while for Bi_2_O_3_@(Pt/Al_2_O_3_) there are no vacant Pt surface sites.

**Figure 3:**
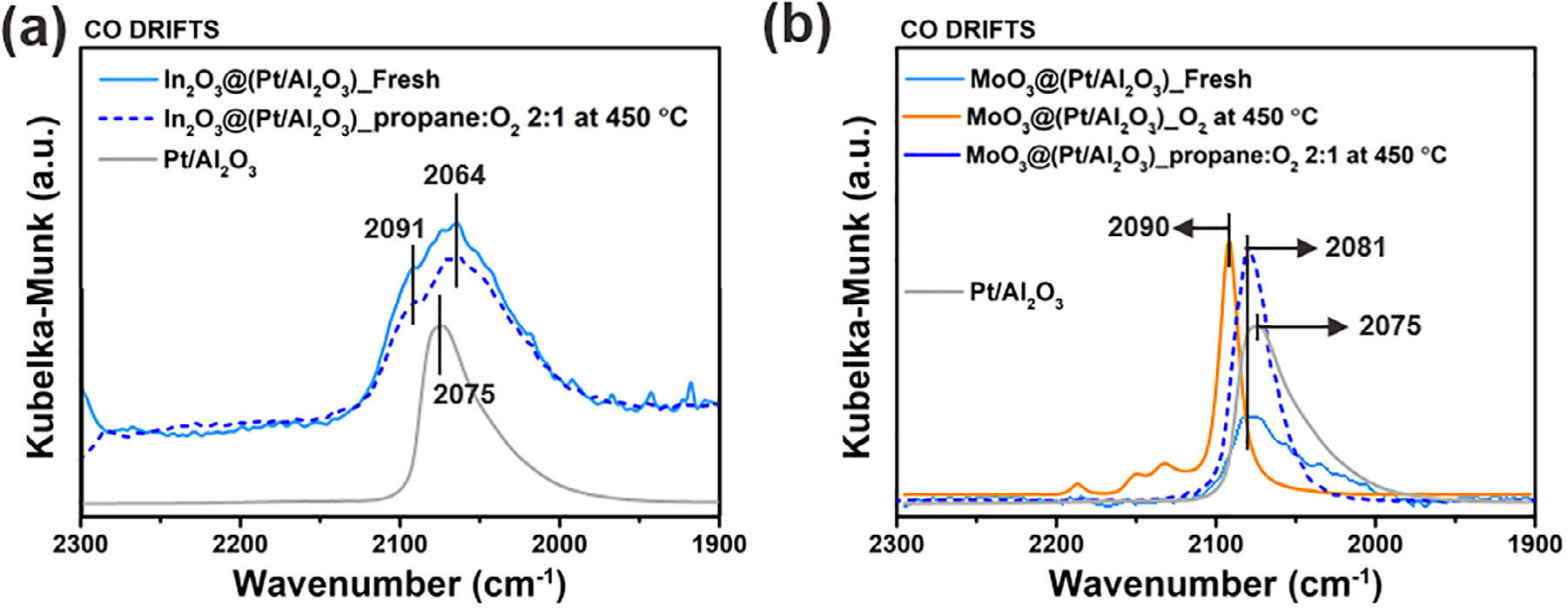
CO DRIFTS spectra of a) In_2_O_3_@(Pt/Al_2_O_3_) and b) MoO_3_@(Pt/Al_2_O_3_) materials after different treatments. Both materials are also compared to Pt/Al_2_O_3_. For In_2_O_3_@(Pt/Al_2_O_3_), fresh and 2:1 propane:O_2_ conditions are shown. For MoO_3_@(Pt/Al_2_O_3_), O_2_ pretreatment is added.

Next, DRIFTS of chemisorbed CO was used to determine if the oxide overcoating altered the types of exposed Pt atoms, and not just the total numbers of exposed Pt determined from pulse chemisorption. The vibrational modes of adsorbed CO report on the different types of surface Pt sites that exist on these materials. In **Figure** **3**, CO adsorbed on Pt/Al_2_O_3_ shows a linear CO adsorption at 2075 cm^−1^ and a tail at low wavenumbers that originates from CO molecules at low‐coordinated Pt sites (kinks, edges, and step‐sites). This spectrum is typical of nano‐sized, multi‐faceted Pt particles on Al_2_O_3_.^[^ ^116^, ^117^ ^]^ In Figure 3a, the In_2_O_3_@(Pt/Al_2_O_3_) material shows two CO peaks, one that is red‐shifted (to 2064 cm^−1^) relative to that of unmodified Pt/Al_2_O_3_, and one that is blue‐shifted (to 2091 cm^−1^). The bands indicate the creation of two new types of Pt domains: relatively electron‐rich (Figure S7a, Supporting Information) and electron‐deficient Pt species.^[^ ^118^ ^]^ The red‐shifted, electron‐rich Pt may indicate small amounts of surface alloying with In.^[^ ^119^ ^]^ The blue‐shifted band at 2091 cm^−1^ indicates partially oxidized Pt at Pt‐In_2_O_3_ interfaces^[^ ^117^ ^]^ and/or hindered CO adsorption due to the OH groups of adjacent In_2_O_3_ surfaces.^[^ ^120^ ^]^ The two new types of Pt confirm the formation of In_2_O_3_‐modified Pt domains and possibly some PtIn alloy. These features are preserved even after being exposed to 450 °C in propane/O_2_, consistent with the stable In_2_O_3_‐Pt interfaces inferred from CO chemisorption.

The CO DRIFTS of as‐synthesized MoO_3_@(Pt/Al_2_O_3_) shows a 2081 cm^−1^ peak, slightly blue‐shifted from that of the parent Pt/Al_2_O_3_, with poor signal‐to‐noise (Figure 3b). The poor signal is consistent with the near‐total loss of accessible Pt seen in the CO chemisorption experiment. After high‐temperature treatment in O_2_, there is a large blue‐shift to 2090 cm^−1^ and an increase in the signal intensity, indicating that Pt sites become uncovered and that they are oxidized Pt sites at the metal‐oxide interface.^[^ ^117^ ^]^ After treatment at 450 °C in 2:1 propane: O_2_, the peak shifts back to 2081 cm^−1^. This is still blue‐shifted relative to native Pt/Al_2_O_3_, indicating CO adsorption on electron‐deficient Pt species (Figure S7a, Supporting Information) that are interacting with adjacent MoO_x_.

**Figure 4:**
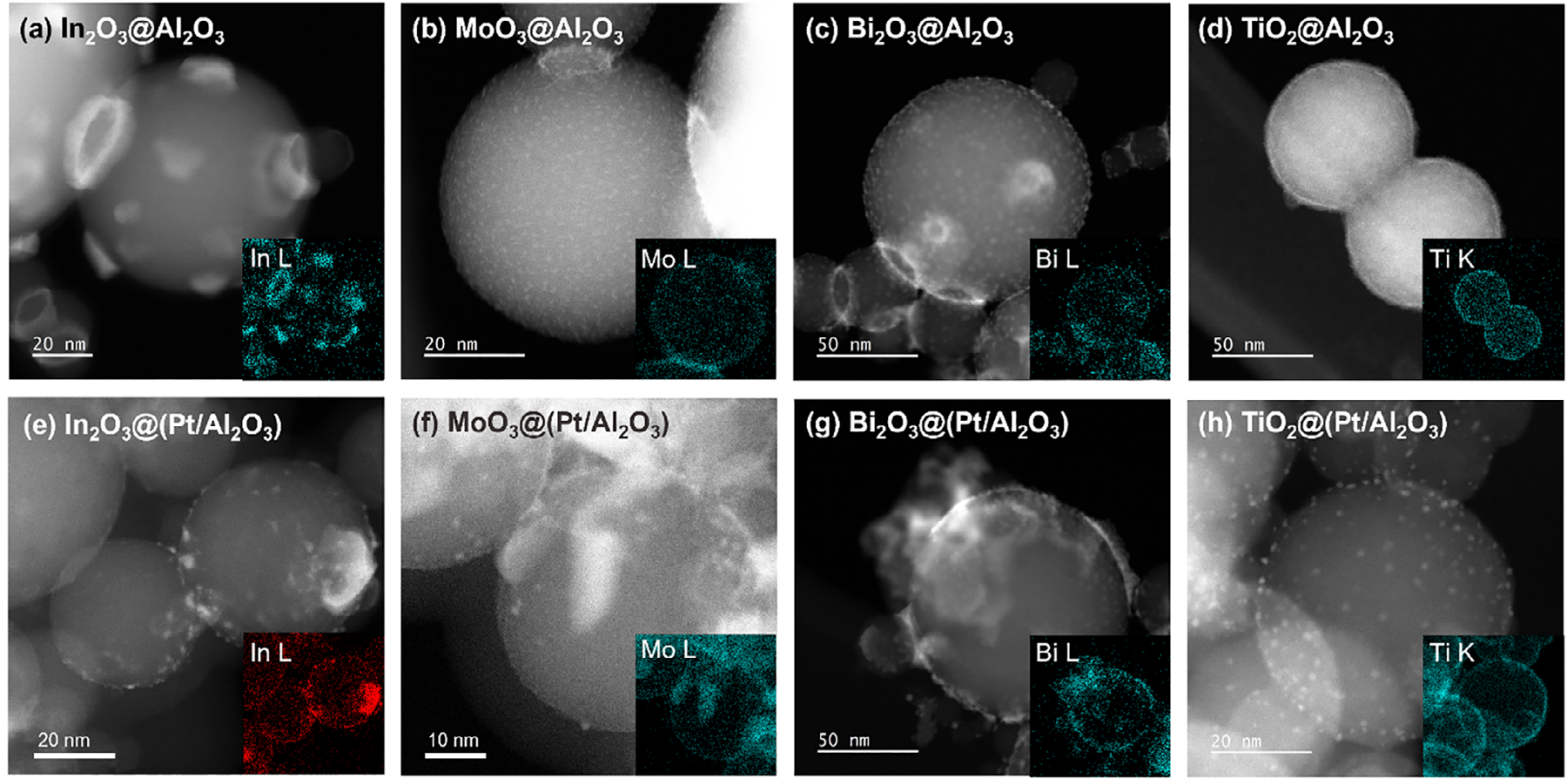
STEM images of the ALD‐overcoated nanomaterials after 450 °C in 2:1 propane: O_2_. Inset EDS maps for overcoating elements (In, Mo, Bi, and Ti). (top row) Al_2_O_3_@MOx, where M is In a), Mo b), Bi c), and Ti d), and (bottom row) (Pt/Al_2_O_3_)@MOx where M is In e), Mo f), Bi g), and Ti h).

While CO DRIFTS indirectly reports on the oxide surface near the Pt NPs, STEM imaging shows bulk morphological changes to the overcoats after thermal treatments. In **Figure** **4**a, the conformal In_2_O_3_@Al_2_O_3_ is massively restructured after treatment at 450 °C in 2:1 propane: O_2_. The resulting ring structures of the In_2_O_3_ suggest that steam formed during combustion of the propane: O_2_ mixture caused the In_2_O_3_ to aggregate at Al_2_O_3_ particle‐particle interfaces due to capillary forces,^[^ ^71^ ^]^ and it explains the continuous deactivation of In_2_O_3_@Al_2_O_3_ in Figure S2b (Supporting Information). However, for In_2_O_3_@Pt/Al_2_O_3_, the restructuring is much less severe (Figure 4e and Figure S9a–c, Supporting Information). While there certainly is some aggregation, other parts of the surface retain the In_2_O_3_ conformal film, as some of us have seen previously.^[^ ^37^ ^]^ Combined with the CO chemisorption and DRIFTS results, it appears that the Pt NPs in Pt/Al_2_O_3_ help pin the In_2_O_3_ overcoat in place, leading to some exposed Pt as well as extensive Pt‐In_2_O_3_ interaction, even after aggressive heat treatment in 2:1 propane: O_2_.

In Figure 4b, we observe the opposite behavior with MoO_3_ overcoats. After the high‐temperature treatment in 2:1 propane: O_2_, the overcoat does not change further on Al_2_O_3_, consistent with the stable reactivity seen in Figure S2d (Supporting Information). On the contrary, substantial restructuring was shown over MoO_3_@(Pt/Al_2_O_3_) (Figure 4f). The MoO_3_ aggregates into larger, disordered particles, which are presumably responsible for uncovering more of the Pt surface. For Bi_2_O_3_, Figure 4c,g are similar to the corresponding images before heat treatment, confirming the stability of the Bi_2_O_3_ overcoat and supporting our observation of complete Pt encapsulation even after the thermal treatment. Figure 4d, h, and Figure S9d–e (Supporting Information) show well‐distributed TiOx clusters and/or very thin layers, especially apparent in the EDS map. There is evidence of local crystallization (Figure S9f, Supporting Information) after the heat treatment, which can explain the reduced Pt coverage seen in the CO chemisorption results.

The above investigations provide the physicochemical properties of metal‐oxide‐overcoated Al_2_O_3_ and Pt/Al_2_O_3_ nanomaterials. Scheme 2b,c illustrate the key features in each of the oxides: 1) *In_2_O_3_* shows strong physical and chemical interaction with Pt NPs, maintaining partial exposure of Pt even after high‐temperature treatments and leading to catalytic behavior consistent with both metal and oxide operating in tandem; 2) *MoO_3_* has strong interaction with Al_2_O_3_, but it restructures to expose Pt with Pt/Al_2_O_3_; 3) *Bi_2_O_3_* completely covers the Pt and is resistant to restructuring, leading to total suppression of dehydrogenation activity and exclusively combustion chemistry; while 4) *TiO_2_* is highly dispersed but seems to interact less with the Pt NPs.

### 3.3 Reducibility of Metal Oxide Overcoated Al_2_O_3_ and Pt/Al_2_O_3_

**Figure 5:**
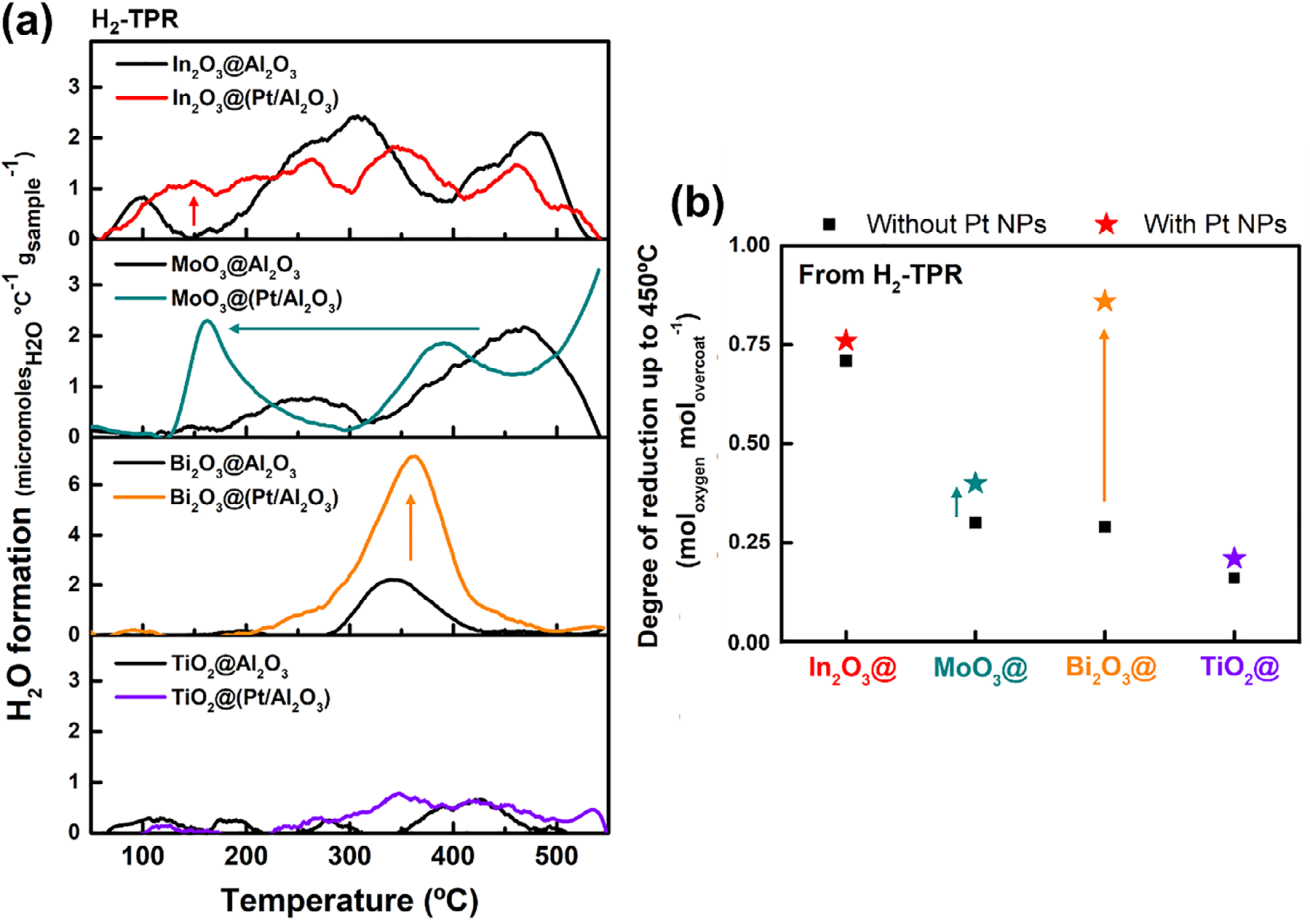
a) H_2_O (m/z = 18) formation profiles during H_2_‐TPR after mild pretreatment at 200 °C under He for 1 h, and b) corresponding degree of reduction (DOR) of ALD overcoated materials with or without underlying Pt nanoparticles. DOR = (mol of H_2_O_evolved_ / mol of M_overcoat_).

**Figure 6:**
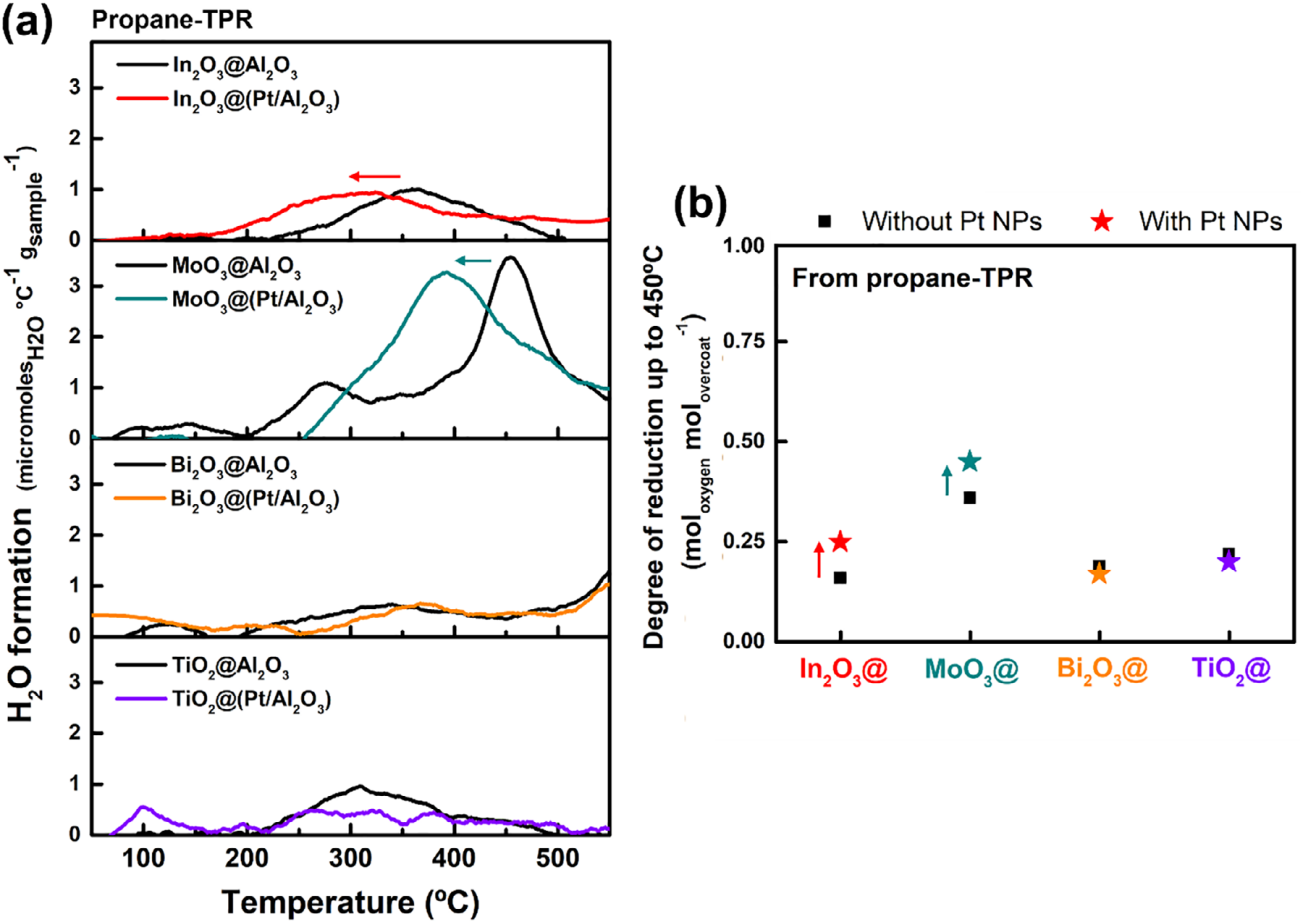
a) H_2_O (m/z = 18) formation profiles during propane (C_3_)‐TPR after mild pretreatment at 200 °C under He for 1 h, and b) corresponding degree of reduction (DOR) of ALD overcoated materials with or without underlying Pt nanoparticles. DOR = (mol of H_2_O_evolved_ / mol of M_overcoat_).

The reducibility of a metal oxide is often correlated with its catalytic activity or utility in other devices, including sensors, capacitors, or batteries.^[^ ^121^, ^122^, ^123^, ^124^, ^125^ ^]^ The properties of very thin overcoated metal oxides can have properties very different from those of the bulk oxides, and underlying Pt nanoparticles are expected to further alter the properties of the overcoats. Reducibility is closely correlated to activity in selective catalytic oxidation ^[^ ^78^, ^126^ ^]^ because the reduction of metal oxide by the reactant (e.g., C_3_H_8_ or H_2_) is typically the rate limiting step in these reactions.^[^ ^127^, ^128^, ^129^ ^]^ To investigate the reducibility of the materials produced, temperature‐programmed reaction (TPR, Figure S10, Supporting Information) was performed in reducing gases (H_2_ or propane (C3)), for the four different overcoated nanomaterials, with or without underlying Pt NPs. An online mass spectrometer was used to selectively measure H_2_O (m/z = 18) evolution as direct evidence of metal oxide reduction (**Figures** **5**a and **6**a; Figures S11 and S12, Supporting Information). We calculated the degree of reduction (DOR) of the metal oxides with or without underlying Pt particles (Figures 5b and 6b; Table S2, Supporting Information) to indicate how much oxygen the oxides can provide under reaction conditions.

During H_2_‐TPR, both In_2_O_3_@Al_2_O_3_ and In_2_O_3_@(Pt/Al_2_O_3_) produce H_2_O starting at ≈60 °C and continue across the entire temperature ramp due to the intrinsic outstanding reducibility of In_2_O_3_ in H_2_ (Figure 5a).^[^ ^69^, ^130^, ^131^ ^]^ Correspondingly, the In_2_O_3_‐based materials show much higher DORs (0.71 and 0.76) than other oxides (Figure 5b), regardless of the presence of Pt. However, the Pt NPs do create a new peak at ≈150 °C, which may be due to activated hydrogen transfer from Pt to adjacent In_2_O_3_. Compared to the other oxides tested here, the TiO_2_ overcoat is poorly reducible and shows marginal changes with Pt NPs (Figure 5a). While there have been reports of Pt NPs improving the reducibility of TiO_2_,^[^ ^132^, ^133^ ^]^ the effect appears to be relatively small for these materials.

In contrast to relatively minor changes for TiO_2_ or In_2_O_3_, underlying Pt NPs significantly enhance the reducibility of MoO_3_ and Bi_2_O_3_ overcoats under H_2_ (Figure 5a). For MoO_3_, the added Pt NPs shift the major peak temperature from 460 °C to 160 °C, while having little impact on the DOR. Recalling that the MoO_3_ overcoat XPS showed little change with Pt, (Figure S6b, Supporting Information), we hypothesize that the improved reduction of MoO_3_ is not due to a change in the MoO_3_ itself, but rather is due to spillover from adjacent Pt.^[^ ^44^, ^134^, ^135^, ^136^, ^137^, ^138^, ^139^ ^]^ Hydrogen spillover is consistent with the proximity of MoO_3_ and Pt domains inferred from CO DRIFTS. For Bi_2_O_3_, underlying Pt NPs substantially increased the H_2_ DOR from 0.29 to 0.86 (Figure 5b), without shifting the peak H_2_O evolution at ≈350 °C (Figure 5a). We correlate the enhanced degree of reduction with the corresponding change in the bulk morphology of the Bi_2_O_3_ in the presence of the buried Pt NPs (Figure 2c, g). The effect of Pt was particularly pronounced after treatment at 450 °C. Bi_2_O_3_@Al_2_O_3_ formed a new species (see XRD in Figure S5c and XPS in Figure S6d, Supporting Information), possibly an Al‐rich Bi‐O‐Al binary phase.^[^ ^109^ ^]^ The resulting material is largely unreducible, while Bi_2_O_3_@(Pt/Al_2_O_3_) remained highly reducible (Figure S13c, Supporting Information). The high reducibility of Bi_2_O_3_@(Pt/Al_2_O_3_) anticipates its high reactivity in propane combustion (Figure S2f,g, Supporting Information) despite the Pt itself being inaccessible (Table 1).^[^ ^126^ ^]^

During propane‐TPR, H_2_O evolution can come either directly from the reduction of the oxide overcoat or by an additional reaction pathway consisting of propane dehydrogenation on Pt, followed by H_2_ spillover. For example, the partial exposure of Pt in In_2_O_3_@(Pt/Al_2_O_3_) and the resulting tandem pathway is proposed to be responsible for the earlier peak of H_2_O evolution (225 °C ves 150 °C, Figure 6a) and the increase in DOR in propane (Figure 6b) since In_2_O_3_ is so readily reduced by H_2_. Conversely, although the TiO_2_ overcoat partially exposes Pt, it does not show obvious H_2_O production during propane‐TPR (Figure 6a). We clearly observe H_2_ generation (m/z = 2) even below 200 °C with TiO_2_@(Pt/Al_2_O_3_) (Figure S12h, Supporting Information), indicative of low temperature propane dehydrogenation over bare Pt, but the TiO_2_ is unable to utilize this H_2_. Bi_2_O_3_ overcoats on either Al_2_O_3_ or Pt/Al_2_O_3_ do not show significant H_2_O formation during propane‐TPR until 500 °C (Figure 6a), and adding Pt results in no significant change in DOR (Figure 6b). Although Bi_2_O_3_ can be reduced by H_2_ at lower temperatures, CO chemisorption showed that the same Bi_2_O_3_ layer completely blocks the Pt and is apparently unable to dehydrogenate propane on its own. These results are consistent with the very low propylene formation rates of Figure 1. Finally, MoO_3_@Al_2_O_3_ shows a strong H_2_O peak at 460 °C during propane TPR, while MoO_3_@(Pt/Al_2_O_3_) shifts the peak to 390 °C and increases the DOR. MoO_3_ itself can dehydrogenate propane (Figure S2d, Supporting Information), but underlying Pt can also contribute by dehydrogenating propane at lower temperatures and spilling the hydrogen over to MoO_3_. Because MoO_3_@(Pt/Al_2_O_3_) can utilize H_2_ at temperatures as low at 160 °C, but H_2_O and H_2_ do not begin to evolve during propane TPR until 250 °C (Figure 6a) or 380 °C (Figure S12d, Supporting Information), respectively, we hypothesize that the MoO_3_ overcoat restructures to expose the Pt NPs at temperatures between 250–380 °C.

We summarize the reducibility results as follows. 1) *In_2_O_3_* has an exposed Pt‐In_2_O_3_ interface that leads to high reducibility in H_2_ and enhanced reducibility in propane via spillover or direct interfaces between Pt and In_2_O_3_. 2) *MoO_3_* completely overcoats Pt, which appears to allow H_2_ activation and spillover for low‐temperature MoO_3_ reduction, but not propane activation, at least not until 250–380 °C when it restructures to expose Pt. 3) *Bi_2_O_3_* also completely overcoats Pt but deposits in a different morphology than on Al_2_O_3_. This morphology has a larger amount of reducible Bi_2_O_3_ exposed and is resistant to forming non‐reducible Bi species; combustion is enhanced, and the reaction of the Pt is completely significantly suppressed. 4) *TiO_2_* leaves exposed Pt, but it remains relatively poorly reducible in H_2_ or C_3_ TPR.

## 4 Conclusion

This study explored the synthesis and properties of inverse structured metal‐metal oxides, termed overcoated materials. Oxides known for catalytic reactivity (In_2_O_3_, MoO_3_, Bi_2_O_3_, and TiO_2_) were deposited on powder Al_2_O_3_ and Pt/Al_2_O_3_ via atomic layer deposition (ALD). We assessed their physicochemical properties (N_2_ physisorption, XRD, and XPS), morphologies (STEM/EDS), interactions with underlying Pt nanoparticles (CO chemisorption and DRIFTS), changes to their reducibility (TPR in H_2_ and propane), and reactivity (propane oxidation). We also examined the effect of thermal pretreatments on the overcoated nanomaterials, which is important for further applications.

The overcoated metal oxides formed either conformal layers or nano‐sized clusters. The few nanometers of overcoats did not generally alter surface area or crystallinity, and the deposited oxides were seen to be in their highest oxidation states (In^3+^, Mo^6+^, Bi^3+^, and Ti^4+^) by XPS after synthesis. In_2_O_3_ and TiO_2_ overcoats partially covered the Pt NPs, while MoO_3_ and Bi_2_O_3_ thoroughly covered them, leaving no accessible Pt sites by CO chemisorption. Thermal treatment restructured the overcoats, exposing Pt sites on the MoO_3_ and TiO_2_ materials. In contrast, In_2_O_3_ and Bi_2_O_3_ demonstrated strong interactions with the underlying Pt NPs, maintaining their as‐synthesized status. In_2_O_3_ has outstanding intrinsic reducibility in H_2_, but the H_2_‐reducibility of the MoO_3_ and Bi_2_O_3_ was notably enhanced when in the form of overcoats on buried Pt NPs. Meanwhile, underlying Pt nanoparticles gave earlier propane‐TPR peak temperatures and higher DOR for the In_2_O_3_‐ and MoO_3_‐based materials, which indicates spillover from Pt to oxide. In_2_O_3_ and Bi_2_O_3_ are known selective hydrogen combustion catalysts via their framework O atoms,^[^ ^78^ ^]^ consistent with their much higher DOR and lower peak temperatures in H_2_‐ than propane‐TPR.

Because In_2_O_3_ is a selective hydrogen combustion catalyst and able to spillover hydrogen from propane dehydrogenation on overcoated Pt nanoparticles, it makes sense that it shows enhanced tandem propane oxidation to propylene (Figure 1a). Conversely, TiO_2_@(Pt/Al_2_O_3_) shows poor reducibility in either propane or H_2_, and its reactivity in propane oxidation is almost the same as Pt/Al_2_O_3_ alone (Figure S2i, a, Supporting Information). Underlying Pt NPs significantly improved the H_2_ reducibility of Bi_2_O_3_, and because the Pt NPs were rendered inaccessible to propane in Bi_2_O_3_@(Pt/Al_2_O_3_), the catalyst is highly selective to total combustion, rather than dehydrogenation. Bi_2_O_3_ has been recently reported to have interesting selective oxidation properties in looping combustion,^[^ ^140^ ^]^ and the material strategies disclosed here may be able to enhance the rates of those materials.

Finally, MoO_3_@(Pt/Al_2_O_3_) activates H_2_ at low temperatures and has plenty of exposed Pt for propane dehydrogenation, but shows no enhanced selectivity toward propylene formation. This must indicate that propane combustion in O_2_ is simply much faster on this catalyst than is the propane dehydrogenation or hydrogen oxidation. MoO_3_@Al_2_O_3_ alone is a poor oxidation catalyst (see Figure S2d, Supporting Information), which supports this hypothesis. The reactivity of this last material lays bare the substantial challenge of matching rates when designing a tandem catalytic system. **Table** **2** summarizes the observed properties of the novel ALD‐overcoated nanomaterials, including morphology, physical interaction between underlying Pt NPs and overcoat, thermal stability of overcoat, and the effect of underlying Pt on the reducibility of overcoat.

**Table 2:** Summary of the physical and chemical properties of ALD‐overcoated nanomaterials.

Overcoated materials	Morphology of the overcoat		Physical interaction between the overcoat and Pt		Overcoat thermal stability	Effect of the underlying Pt on the reducibility of the overcoat
	Fresh	Heat treated	Fresh	Heat treated		
In_2_O_3_@Al_2_O_3_	layer	aggregates	–	–	Poor	Small enhancement in H_2_ and C_3_; H_2_ readily reduces In_2_O_3_
In_2_O_3_@(Pt/Al_2_O_3_)	layer	layer/clusters	Good	Good	Fair	
MoO_3_@Al_2_O_3_	clusters	clusters	–	–	Good	Large enhancement in H_2_; small enhancement in C_3_
MoO_3_@(Pt/Al_2_O_3_)	clusters	aggregates	Great	Fair	Poor	
Bi_2_O_3_@Al_2_O_3_	clusters	clusters	–	–	Good	Large improvement in H_2_ even after thermal treatments
Bi_2_O_3_@(Pt/Al_2_O_3_)	lacey layer	lacey layer	Great	Great	Fair	
TiO_2_@Al_2_O_3_	clusters	clusters	–	–	Good	Marginal effect on reducibility
TiO_2_@(Pt/Al_2_O_3_)	layer	layer	Poor	Poor	Fair	

Our findings highlight novel aspects of inverse structured nanomaterials that become synthetically possible by leveraging layer‐by‐layer deposition of reactive metal oxides. The data provide design principles for overcoated nanomaterials that can potentially be applied as tandem heterogeneous catalysts, materials for chemical looping, sensors, or other functional materials. For example, In_2_O_3_ is reactive for alkane/H_2_ oxidation and CO_2_ activation, MoO_3_ for alkane dehydrogenation, and Bi_2_O_3_ for both alkane dehydrogenation/oxidation and H_2_ oxidation. Additionally, we can modify the reactivity of overcoats by combining them with the many catalytically active metal NPs and/or by using restructuring behaviors under different conditions. As a result, this study on overcoated nanostructures provides novel insights into engineering functional tandem nanomaterials.

## Conflict of Interest

The authors declare no conflict of interest.

## Supporting information



Supporting information

## Data Availability

The data that support the findings of this study are available from the corresponding author upon reasonable request.
