# Supplementary material for: Pt/Al2O3 Overcoated with Reactive Metal Oxides and Their Application to Catalytic Oxidation of Propane
Source: Small Methods. 2025 Oct 1;9(11):e01377. doi: 10.1002/smtd.202501377 (PMC12641361; doi:10.1002/smtd.202501377)
Supplement: Supplementary file 1 — Supporting information [file SMTD-9-e01377-s001.pdf]

Supporting Information for:

Pt/Al_2O_3 Overcoated with Reactive Metal Oxides and Their Application to Catalytic Oxidation of Propane

Geun-Ho Han[a,b], Kunmo Koo[c], Selim Alayoglu[b], Siobhan W. Brown[b], Justin M. Notestein[a,b,*]

[a] Department of Chemical and Biological Engineering, Northwestern University, 2145 Sheridan Road, Evanston, Illinois 60208, United States

[b] Center of Catalysis and Surface Science, Northwestern University, 2145 Sheridan Road, Evanston, Illinois 60208, United States

[c] NUANCE, Northwestern University, 2145 Sheridan Road, Evanston, Illinois 60208, United States

*Corresponding author. E-mail: j-notestein@northwestern.edu (Justin M. Notestein)

1. Catalyst preparation

1.1. Pt/Al_2O_3 preparation methods

Pt/Al_2O_3 catalyst was prepared by wet impregnation method using a rotary evaporator (BUCHI) equipped with a vacuum pump. Six grams of sieved (53–150 µm, Dual MFG) Al_2O_3 (spherical Al_2O_3 nanoparticles, NanoArc, Alfa Aesar) was dispersed in 100 mL of DI water (18.2 MΩ cm^{-1}, Nanopure, Thermo Scientific) contained in a single neck round bottom flask that is compatible with the evaporator. 120 mg of tetraamineplatinum(II) nitrate ($(NH_3)_4Pt(NO_3)_2$, Thermo Scientific) was dissolved in 4 mL of DI water and the resultant solution was added to the Al_2O_3 containing suspension. The amount of Pt precursor corresponds to 1 wt.% Pt on Al_2O_3, and the resultant had 0.8 wt.% Pt measured by ICP-OES. The flask containing all components was connected to the evaporator and the temperature of the water bath for the flask was set to 50°C. After rotating and evaporating overnight, the resultant powder was collected and sieved again (53–150 µm), followed by calcination at 300°C for 4 hours in static air in a muffle furnace (Thermolyne, Thermo Scientific). This temperature was deemed sufficient from an initial thermogravimetric analysis (TGA) profile of the as-synthesized materials.

1.2. $MOx@Al_2O_3$ and $MOx@(Pt/Al_2O_3)$ (M = In, Mo, Bi, and Ti) by atomic layer deposition (ALD)

The Al_2O_3 support was used for ALD without any further treatments except for sieving (53–150 µm), and Pt/Al_2O_3 was used after the mild calcination indicated above. To deposit an overcoat material, the same ALD recipe was applied to both Al_2O_3 and Pt/Al_2O_3. Three similar versions of ALD instruments (GEMSTAR ALD SYSTEM (Arradiance, in the GaintFab facility at Northwestern University), GEMSTAR 8 REMAN SYSTEM (Arradiance, in the GaintFab

facility at Northwestern University), and GEMSTAR XT^{TM} (Arradiance, in the NUFAB facility at Northwestern University)) were used with a powder sample holder: two 316 stainless steel wire cloths (McMaster-Carr, 500 × 500 mesh, opening size of ~ 30μm) sandwiched the powder sample, and they were put on a thin steel plate, which is followed by assembling steel top and bottom holder parts with six screws to lock up the powder holder. All ALD instruments are equipped with an Ozone Gas Generator (Pacific Ozone) to supply O_3 as an oxygen source. The GEMSTAR XT^{TM} instrument was equipped with a power supply (SEREN, R301MK11 RF power supply) to apply O_2 and Ar plasma to the chamber. Any given instrument was used to synthesize all materials of a given elemental composition.

To more efficiently deposit In on Al_2O_3 and Pt/Al_2O_3, the method in the previous study was modified. Yan et al. used cyclopentadienylindium(I) (InCp) as an In precursor and ozone (O_3) as an oxygen supply, and about 35 ALD cycles were required to deposit about 7.5 wt.% of In.[1] To enhance the growth rate of indium oxide layers, the chamber temperature was elevated from 150°C to 225°C, and the combination of O_3 and H_2O were employed together, because the dual oxidants were found to provide a more effective oxidation process compared to the individual species.[2] In the meantime, the InCp bottle temperature was fixed at 50°C because temperatures of 60°C or higher caused severe unreversible clogging. As a result of the modification, only 4 cycles of ALD cycles can deposit about 7 wt.% of In on both Al_2O_3 and Pt/Al_2O_3 (**Table 1**).

To deposit Mo on Al_2O_3 and Pt/Al_2O_3, an O_2-plasma enhanced atomic layer deposition (PEALD) was modified.[3] Plasma-derived radicals can facilitate the oxidation of organic compounds, enabling lower temperature ALD. Compared to prior studies, the chamber temperature was lowered from 200°C to 150°C to obtain more conformal MoOx layers. As a result, 25 ALD cycles deposits about 6 wt.% of Mo on the powder substrates.

For Bi deposition, triphenylbismuth ($BiPh_3$) was used as a Bi precursor and O_3 was employed as an oxygen supply.[4] Compared to other bismuth precursors (*e.g.*, $Bi(OCMe_2Et)_3$, $Bi(thd)_3$, $BiMe_3$, or $BiEt_3$), $BiPh_3$ has better volatility and stability, and is moderately sensitive to air and moisture.[4–6] Indeed, $Bi(thd)_3$ was not a successful ALD precursor in our hands. Using $BiPh_3$ under conditions described in **Table 1**, Bi deposition of about 13 wt.% was achieved in 10 ALD cycles.

For Ti deposition, titanium(IV) isopropoxide (TTIP) and H_2O were used as a Ti precursor and an oxygen supply, respectively. The temperatures of the bottle, manifold, and chamber were set to 80, 115, and 250°C.[7] Under the conditions in **Table 1**, 100 cycles of Ti ALD resulted in about 3 wt.% of Ti on the supports.

2. Material characterization

N_2 adsorption-desorption isotherms were obtained at 77K by using a Micromeritics 3FLEX instrument in the REACT core facility at Northwestern University. Before the analysis, materials were degassed under a dynamic vacuum (<5 mm Hg) at 150°C overnight. The collected isotherm data set was analyzed by the Brunauer–Emmett–Teller (BET) equation and the Non-Local Density Functional Theory (NLDFT) model to calculate specific surface area (S_{BET}) and pore information, respectively. The slit-NLDFT model matched the H3 type isotherm indicating its origin from interparticle regions rather than internal pores.

Temperature-programmed reduction by hydrogen (H_2-TPR) or propane (C_3-TPR) was performed using an AutoChem II 2920 (Micromeritics) with an online mass spectrometer (SRS Universal gas analyzer, UGA) in the REACT core facility at Northwestern University. The AutoChemII was used to obtain thermal conductivity detector (TCD) signals and individual mass spectra of effluents such as hydrogen (H_2), water (H_2O), carbon dioxide (CO_2), propylene (C_3H_6), and propane (C_3H_8) during TPR. Materials were packed into a U-type quartz tube with

quartz wool and silicon carbide used to maintain the height and volume of the material bed in the tube. For both TPRs, two pretreatments were employed; 200°C and 450°C under 20 sccm of He for 1 hour to compare a mild pretreatment with one typical of the oxidative dehydrogenation of propane reaction.[1] The pretreatments were followed by TCD and MS signal stabilization steps and the material temperature was ramped to 550°C at 10°C/min under 40 sccm of 4% H_2/Ar, or 10% propane/Ar. MS data quantification was implemented by using sensitivity factors for each effluent gas, followed by normalization and calculation to measure the actual amounts (moles) of gas components in the effluent. The degree of reduction (DOR) was defined as the moles of H_2O evolved until 450°C, divided by the total number of metal atoms in the overcoat (In, Mo, Bi, or Ti) from ICP-OES analysis. DOR is expressed as an absolute value and is not normalized for the stoichiometry of the bulk oxide (*i.e.*, In_2O_3 vs. Bi_2O_3 vs. TiO_2 vs. MoO_3,).

Carbon monoxide (CO) pulse chemisorption was performed using the AutoChem II 2920 (Micromeritics) in the REACT core facility at Northwestern University to measure the amount of CO adsorption, and from that, the average particle sizes of Pt on the material. Following the same sample loading procedures from TPR, samples were pretreated at 200°C under 20 sccm 4% H_2/Ar for 1 hour. Then, the material was cooled to 45°C, and pulse chemisorption was conducted, assuming a 1:1 CO:Pt_{surf} adsorption stoichiometry.

Transmission electron microscopy (TEM) and scanning transmission electron microscopy (STEM) images were taken in the NUANCE facility at Northwestern University. Material particles are dispersed in methanol (Fisher Scientific, ≥99.8%) and drop-casted on the lacey carbon TEM grid (Ted Pella). The sample is dried under an infrared lamp. All S/TEM and EDS analysis is obtained with a JEOL ARM200CF probe-corrected field-emission transmission electron microscope. The acceleration voltage is 200 keV and the probe diameter is 1 Å with 62 pA of probe current at the STEM imaging condition. Two silicon drift detectors

(SDD) from the JEOL having a total acceptance solid angle of 1.5 srad are used.

Diffuse reflectance infrared Fourier transform spectroscopy (DRIFTS) using carbon monoxide as a probe molecule was implemented by a Thermo Nicolet 6700 FTIR spectrometer equipped with a praying mantis diffuse reflectance sample holder in the REACT core facility at Northwestern University. Materials were loaded inside the sample holder without dilution. Samples were pretreated at 200°C under 100 sccm of ultra-high pure Ar for 1 hour, cooled to 40°C, and backgrounds were acquired. 100 sccm of 5% CO/Ar was fed into the holder until the gas phase CO vibrational bands were saturated. Then, the holder was flushed with 100 sccm of Ar to remove the remaining CO molecules, and the CO vibrational bands on the material surface were obtained by subtracting the background. The diffuse reflectance spectra were acquired by the average of 64 scans with a resolution of 4 cm^{-1} and then the Kubelka - Munk transform ($KM = (1-R)^2/2R$, where R is Rs/Rb) was applied to the spectra with the OMNIC FTIR software (Thermo Scientific).

X-ray photoelectron spectra (XPS) were collected by using a NEXSA G2 (Thermo Scientific^{TM}, Al Kα radiation, 1486 eV) with an electron flood gun (non-conductive materials) in the NUANCE facility at Northwestern University. After the acquisition, spectra were calibrated to the C 1s (284.6 eV). If the Y-axis is presented as normalized intensity, the spectra were normalized by the maximum height to compare.

Quantification of platinum (Pt), indium (In), aluminum (Al), titanium (Ti), bismuth (Bi), and molybdenum (Mo) was accomplished using ICP-OES of acid-digested samples in the QBIC center at Northwestern University. Samples were digested in 1.5 mL concentrated trace-grade nitric acid (> 69%, Thermo Fisher Scientific, Waltham, MA, USA) and 1.5 mL concentrated trace-grade hydrochloric acid (> 34%, Thermo Fisher Scientific, Waltham, MA, USA) and microwaved using a custom method including a 20-minute linear ramp to 180°C, a

30-minute hold at 180°C, and 15 minutes of exhaust time, using a CEM Mars6 microwave digestion system. Samples containing Mo or Ti then had 2 mL of a 5% hydrofluoric acid solution added (5% HF, a 10 times dilution of product 87003-257, 51%, ARISTAR PLUS for trace metal analysis, VWR Chemicals BDH) and were microwaved a second time using the same method. The digested solutions were transferred to pre-weighed 50 mL metal-free falcon tubes and triple-rinsed with ultra-pure H_2O (18.2 MΩ·cm) to ensure a quantitative transfer and to produce a solution in 45 mL total. Quantitative standards were prepared by diluting individual 1,000 ug/mL elemental standards (Inorganic Ventures, Christiansburg, VA, USA) in a matrix of 2% nitric acid and 2% hydrochloric acid (v/v). ICP-OES was performed on a computer-controlled (QTEGRA software) Thermo iCap7600 ICP-OES (Thermo Fisher Scientific, Waltham, MA, USA) operating in radial view for In, Ti, and Al wavelengths and axial view for Pt, Mo, and Bi wavelengths, and equipped with an ESI-SC-2DX PrepFAST autosampler (Omaha, NE, USA). Each sample was acquired using a 5-second visible exposure time and 15-second UV exposure time, running 3 replicates. The spectral lines selected for analysis were: Al (396.152, 309.271 nm), In (325.609, 303.936 nm), Pt (214.423, 203.646, 224.552 nm), Mo (202.030, 204.598, 203.844 nm), Ti (334.941, 323.452, 336.121 nm), Bi (223.061, 190.234 nm).

The powder X-ray diffraction (PXRD) patterns were collected by a STOE-STADI P diffractometer by using CuKα1 radiation in the range between two theta of 20°-80°. Using the equipped auto-sampler and the powder sample holders, the series of powder materials were analyzed.

3. Propane oxidation reaction in a fixed-bed reactor

Catalytic performance tests of propane oxidation were implemented in a U-type quartz tube reactor. 100 mg catalyst was diluted in a mass fraction of catalyst/diluent = 1/4 with quartz

sand (Sigma-Aldrich) to minimize mass transfer limitations.[8] To locate the catalyst at the same level, the diluted catalyst was placed with quartz wool at a funnel-like bottom of the reactor made from two different inner diameters of the U-type tube. The void volume after the catalyst bed was filled with quartz beads to suppress gas-phase reactions of hydrocarbons.[9] An aluminum block heater is connected to a heating controller with software (J-KEM). Reactant gases are 20% propane in He and 10% O_2 in He. Two mass flow controllers were connected to the two reactant gases, and the other to ultra-pure He for pretreatment. 5 sccm of each gas (GHSV = 600 $sccm_{C3}\ g_{cat}^{-1}\ h^{-1}$) controls the stoichiometry as $C_3H_8/O_2 = 0.5$. Before the reaction tests, catalysts were pretreated at 450°C under He for 1 hour, followed by 6 hours of performance tests after switching the He gas to the reaction mixture. An online connected gas chromatograph (Shimadzu, GC-2014) was used for analyzing the products. A Carboxen 1010 PLOT (length of 30 m and inner diameter of 0.53 mm) was connected to a thermal conductivity detector (TCD) to analyze O_2, CO, and CO_2, and a GS-Gaspro (length of 30 m and inner diameter of 0.32 mm) column to a flame ionization detector (FID) to analyze hydrocarbons (*i.e.*, methane, ethane, ethylene, propane, and propylene).

4. Supporting Schemes, Tables, and Figures

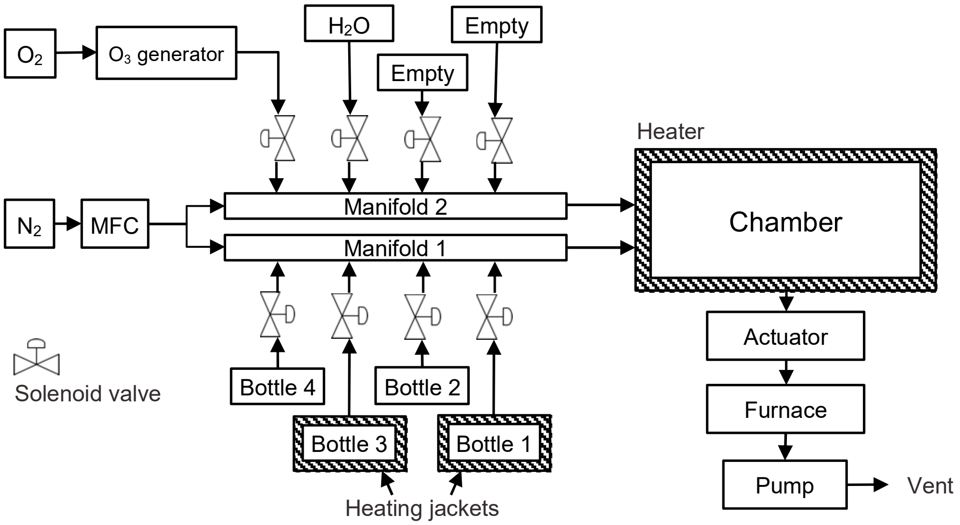

Scheme S1. Schematic of lab-scale atomic layer deposition system. Eight ports can be connected to a bottle that contains a source (metal or O). Each manifold is connected to four ports. Both manifolds are connected to a chamber. A sample is placed inside the chamber to implement ALD. The exhaust line from the chamber is connected through a furnace at 550°C to combust unreacted ALD precursors, a high-functional vacuum pump, and a vent. One of the ports is connected to the O_3 generator to supply O_3.

Table S1. Textural properties of parent materials and ALD-overcoated nanomaterials.

Materials	Specific surface area by BET equation (m^2 g_{sample}^{-1}, S_{BET})	Pore volume by NLDFT method (cm_3 g^{-1})
Al_2O_3	40.3	0.097
(Pt/Al_2O_3)	38.7	0.190
$In_2O_3@Al_2O_3$	32.7	0.080
$In_2O_3@(Pt/Al_2O_3)$	35.1	0.194
$MoO_3@Al_2O_3$	32.1	0.065
$MoO_3@(Pt/Al_2O_3)$	28.9	0.145
$Bi_2O_3@Al_2O_3$	33.7	0.095
$Bi_2O_3@(Pt/Al_2O_3)$	32.4	0.189
$TiO_2@Al_2O_3$	37.2	0.074
$TiO_2@(Pt/Al_2O_3)$	31.5	0.145

Table S2. Degree of reduction (DOR) at 450°C for as-synthesized and thermally-treated ALD-overcoated nanomaterials.

Materials	DOR[a] for as-synthesized (mol_{oxygen} $mol_{overcoat}^{-1}$)		DOR for thermally-treated (mol_{oxygen} $mol_{overcoat}^{-1}$)[b]	
	H_2-TPR	Propane-TPR	H_2-TPR	Propane-TPR
In_2O_3@Al_2O_3	0.71	0.16	0.22	0.14
In_2O_3@(Pt/Al_2O_3)	0.76	0.31	0.32	0.46
MoO_3@Al_2O_3	0.30	0.36	0.24	0.25
MoO_3@(Pt/Al_2O_3)	0.40	0.45	0.37	0.81
Bi_2O_3@Al_2O_3	0.29	0.19	n.d.	0.08
Bi_2O_3@(Pt/Al_2O_3)	0.86	0.17	0.82	0.20
TiO_2@Al_2O_3	0.16	0.29	0.11	0.12
TiO_2@(Pt/Al_2O_3)	0.21	0.20	0.14	0.18

[a)] DOR = (mol of $O_{in\ H_2O}$ / mol of $M_{overcoat}$). The total number of O in the produced H_2O until 450°C TPR from mass spectrum divided into the total number of overcoating metals (In, Mo, Bi, and Ti) from ICP-OES analysis. No normalization for metal oxide stoichimetry was made.
[b)] 450°C in He for 1 hour treatment.

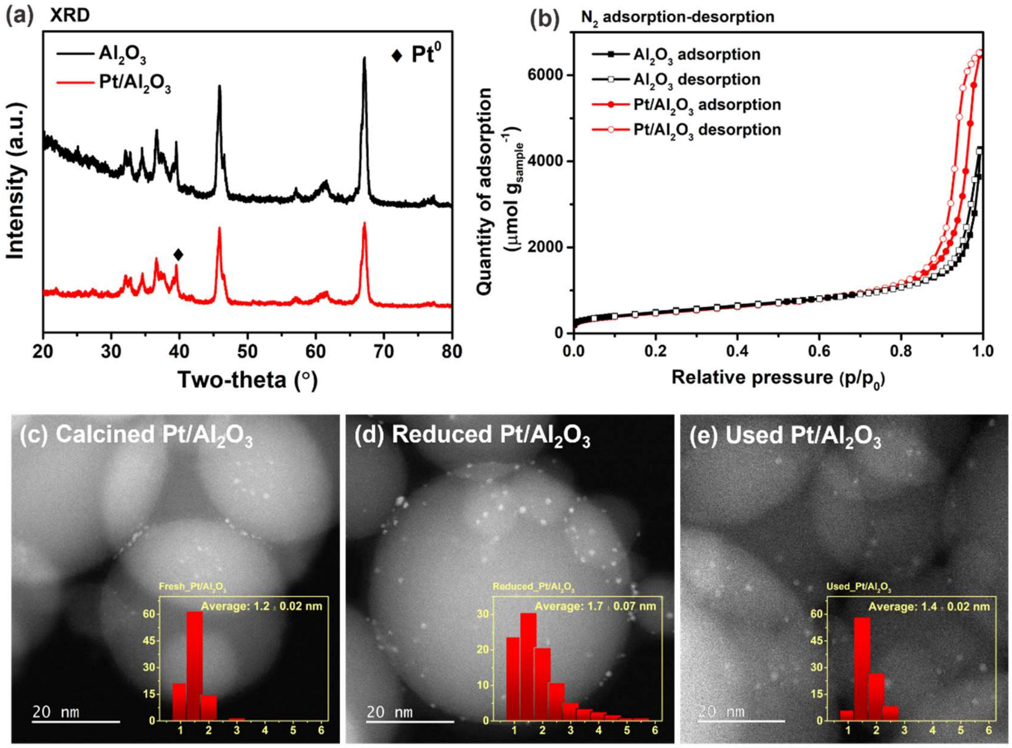

Figure S1. (a) XRD patterns for Al_2O_3 and Pt/Al_2O_3. The location of a representative Pt(111) feature is marked, but is not observed in the XRD data. (b) N_2 adsorption-desorption isotherms of Al_2O_3 and Pt/Al_2O_3. STEM images for (c) calcined, (d) reduced, and (e) 450°C 2:1 propane:O_2 treated Pt/Al_2O_3. Inset figures show particle size distributions with average particle sizes.

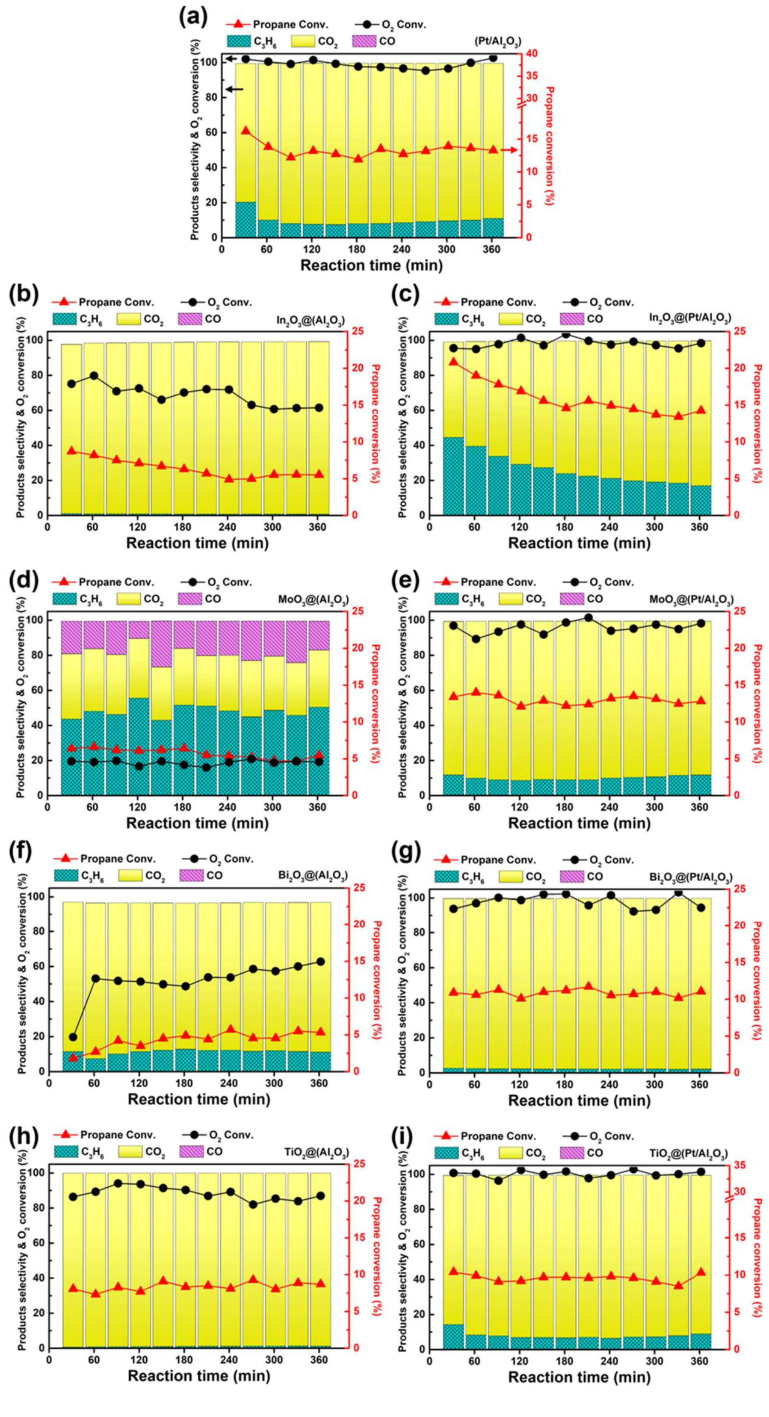

Figure S2. Catalytic performance in the oxidation of propane. Conditions: 450°C 1 h under He, then GHSV 600 $sccm_{C3}$ g_{cat}^{-1} h^{-1}, with 10% propane and 5% O_2, balance He. Left y-axis: product selectivity (cumulative bar graph; cyan propylene, yellow CO_2, and pink CO) and O_2 conversion (black circles). Right y-axis: propane conversion (red triangles) (a) Pt/Al_2O_3, (b) In_2O_3@Al_2O_3, (c) In_2O_3@(Pt/Al_2O_3), (d) MoO_3@Al_2O_3, (e) MoO_3@(Pt/Al_2O_3), (f) Bi_2O_3@Al_2O_3, (g) Bi_2O_3@(Pt/Al_2O_3), (h) TiO_2@Al_2O_3, and (i) TiO_2@(Pt/Al_2O_3).

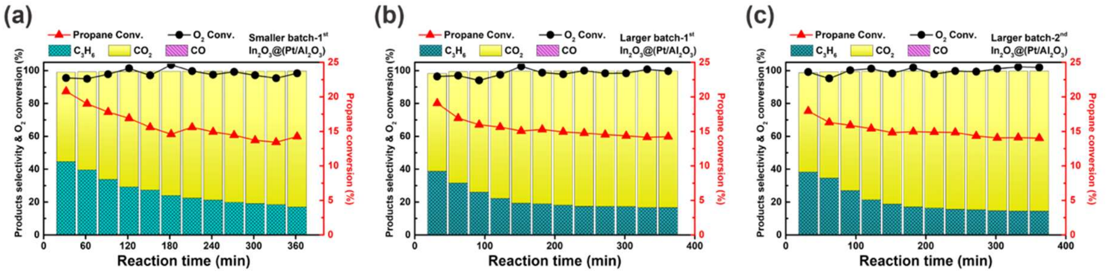

Figure S3. Reproducibility test for In_2O_3@(Pt/Al_2O_3) nanomaterial. (Left, a) Reproduced from Figure S2. (b) A new batch of In_2O_3@(Pt/Al_2O_3) nanomaterial was synthesized at twice the scale of the original batch (150 mg to 300 mg) and retested. (c) A third batch was synthesized and retested, at the same scale as for (b). The similar results for all three indicates the reproducibility of ALD and overcoated nanomaterials for lab-scale research.

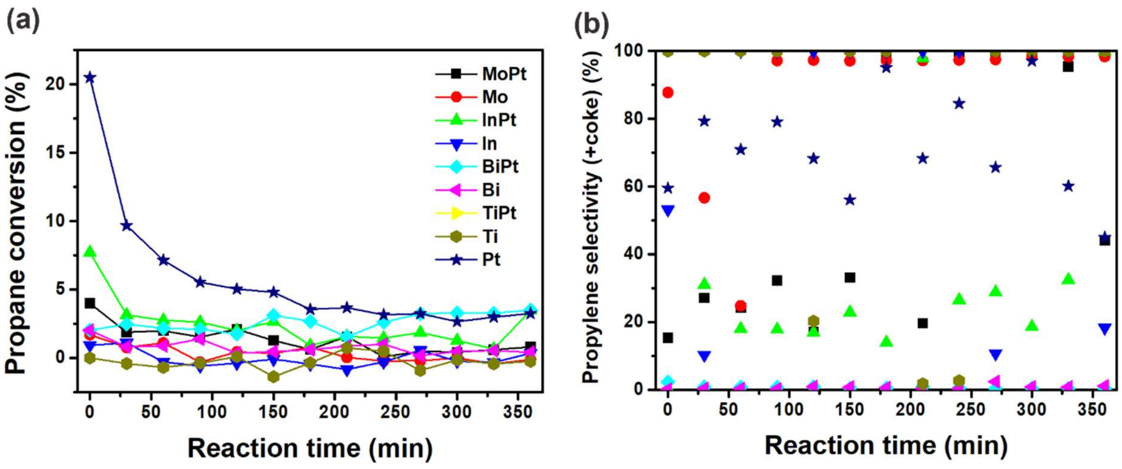

Figure S4. Catalytic test results of propane (non-oxidative) dehydrogenation. Conditions: pretreatment at 450°C for 1 h under He, followed by GHSV 600 $sccm_{C3}$ g_{cat}^{-1} h^{-1} of 20% propane in He. (a) Propane conversion with reaction time. (b) Total (including coke formation), carbon-based propylene selectivity. Among gaseous products, most propylene selectivities were close to 100%, however many catalysts predominantly formed coke.

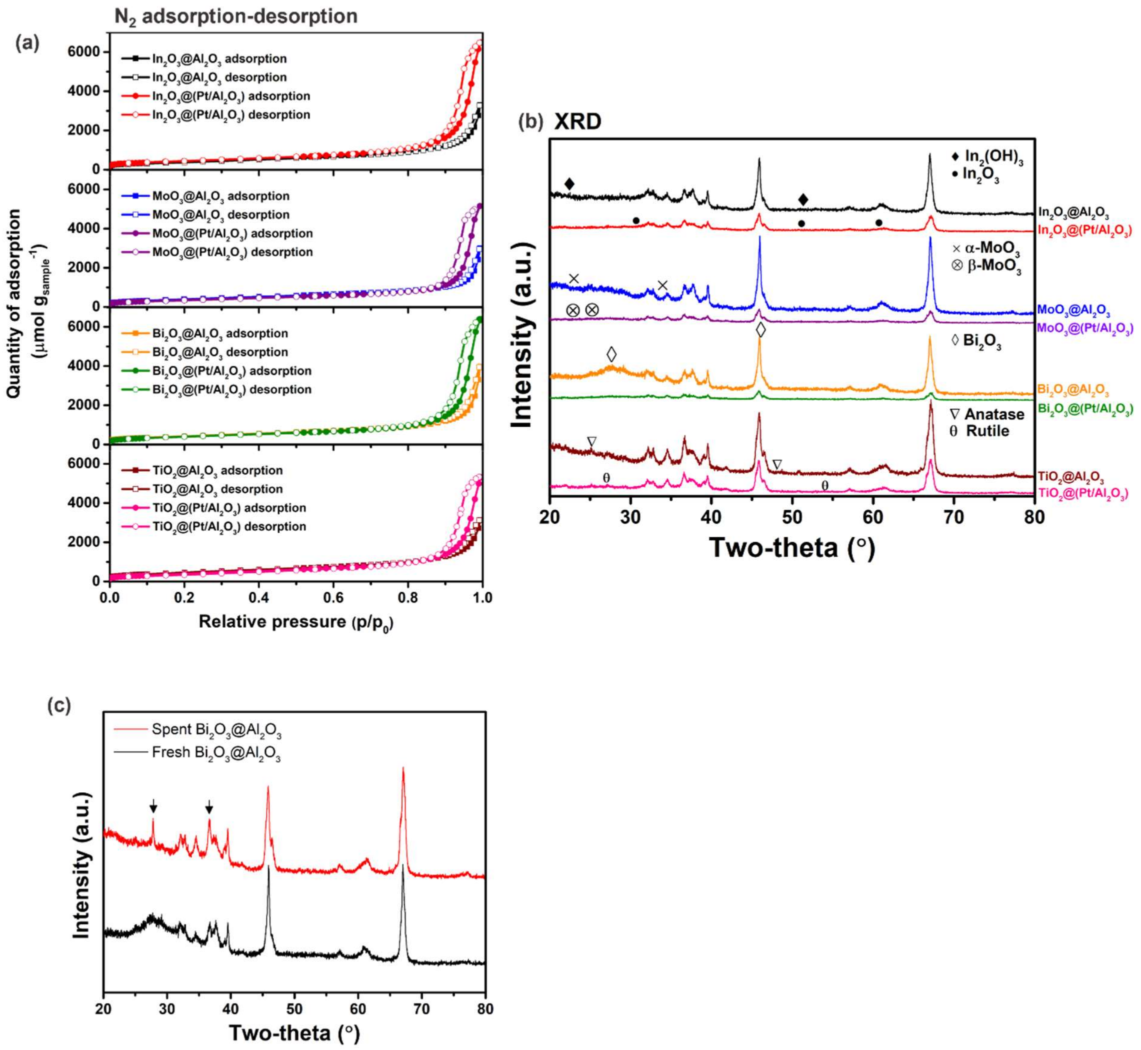

Figure S5. (a) N_2 adsorption-desorption isotherms of ALD-overcoated nanomaterials. Solid and open symbols represent adsorption and desorption, respectively. (b) XRD patterns of ALD-overcoated nanomaterials. Symbols indicate the location of crystalline oxides, although none are distinctly formed here. All peaks are from the Al_2O_3 support; see Figure S1. (c) XRD patterns of fresh and spent Bi_2O_3@Al_2O_3. In the spent material, a new peak is observed near 27°, which can be assigned as Bi_2O_3 (210).[10] There is no evidence to indicate the formation of $Bi_2Al_4O_9$ (expected features at 15° and 30°)[11] or $BiAlO_3$ (expected features at 22° and 45°).[12]

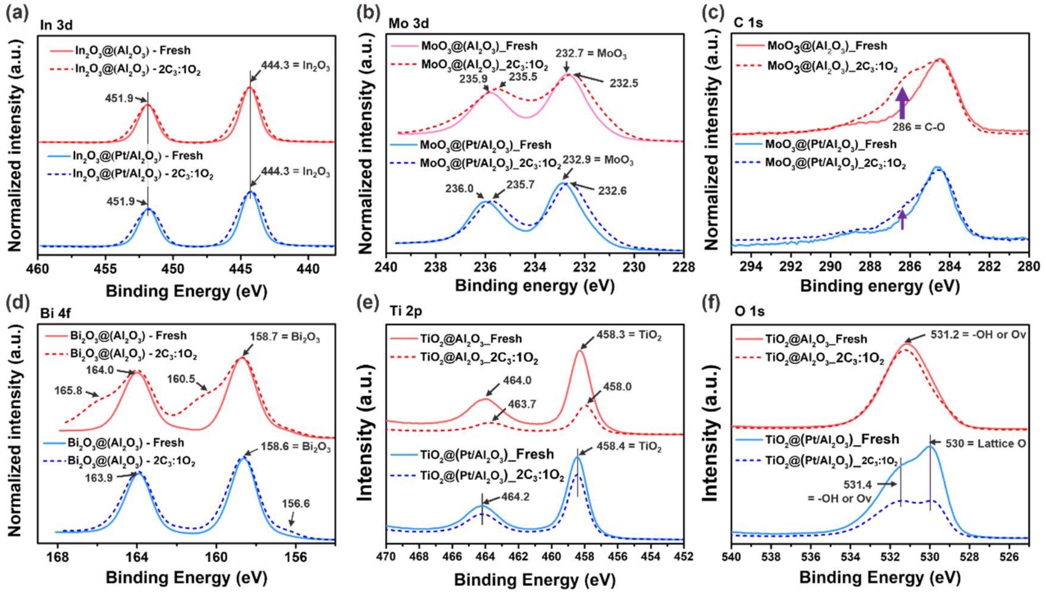

Figure S6. XPS spectra of overcoated nanomaterials. (a) In 3d region for In_2O_3-overcoated ones, (b) Mo 3d region for MoO_3-overcoated ones, (c) C 1s region for MoO_3-overcoated ones, (d) Bi 4f region for Bi_2O_3-overcoated ones, (e) Ti 2p region for TiO_2-overcoated ones, and (f) O 1s region for TiO_2-overcoated ones.

As-synthesized oxidation states are compared to literature.[13–20] Both In_2O_3 overcoated materials show two peaks at 451.9 and 444.3 eV, which are assigned to In^{3+}. Both MoO_3 overcoated materials show peaks at 235.9–236.0 eV and 232.7–232.9 eV which are assigned to Mo^{6+}. In the Bi 4f region, peaks observed at 163.9–164.0 and 158.6–158.7 eV originate from Bi^{3+}. In the Ti 2p region, 464.0–464.2 and 458.3–458.4 eV peaks are attributed to Ti^{4+}. **Figure S6f** shows dominant peaks at 531.4 assigned to the hydroxyl groups, indicating numerous surface defects.

MoO_3-based and Bi_2O_3-based materials show changes in the XPS spectrum after treatment at 450°C in 2:1 propane:O_2 (dotted lines). For Mo, peaks shift to lower binding energies (0.3–0.5 eV), indicating partial to Mo^{5+}. **Figure S6d** shows the evolution of new electronic states for $Bi_2O_3@Al_2O_3$ after reaction at 165.8 eV for Bi 4f $_{5/2}$ and 160.5 eV for Bi 4f $_{7/2}$. There have been several claims made for the origin of these highly-oxidized electronic states. A charging effect was suggested,[13,21] but that seems doubtful in this case. $Bi_2Al_4O_9$ has been reported to have lower binding energies than Bi_2O_3, opposite to our case.[22] Depending on the dispersion of BiOx on Al_2O_3, either (Bi-rich) or (Al-rich) Bi-O-Al binary phases could be formed.[15] Bi_2O_5 (Bi^{5+}) has similar binding energies,[23,24] but it is not clear how such a structure would form in this case. Meanwhile, the monolayered Bi_2WO_6 nanosheet showed peaks at 165.0 eV for Bi $4f_{5/2}$ and 159.9 eV for Bi $4f_{7/2}$, similar to those seen here.[14]

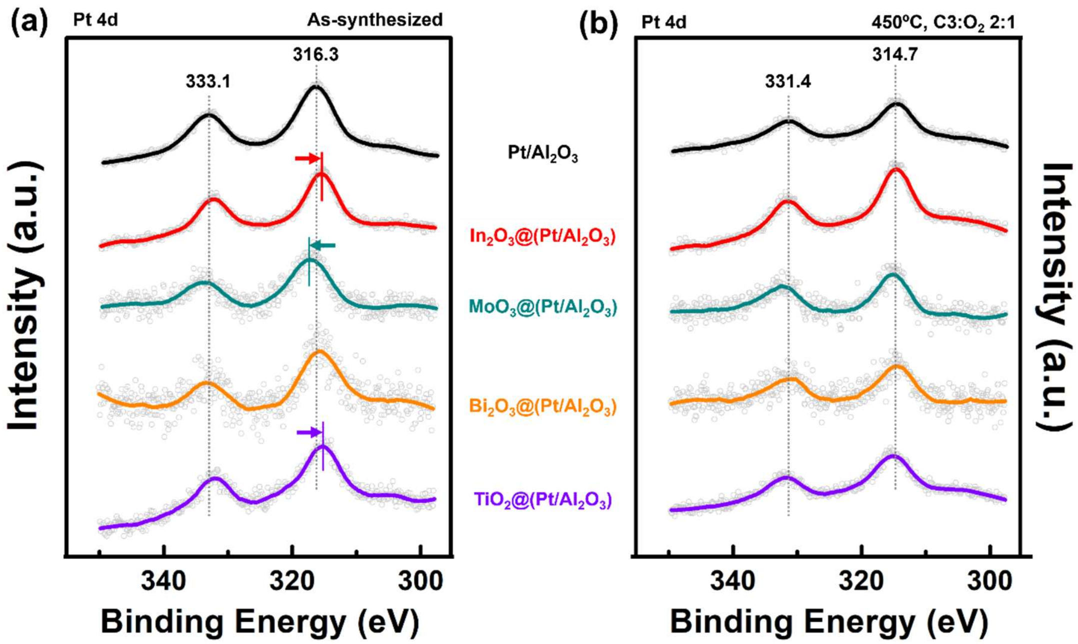

Figure S7. Pt 4d XPS spectra for Pt/Al_2O_3 and ALD-overcoated Pt/Al_2O_3 nanomaterials. (a) as-synthesized materials and (b) after reaction at 450°C, propane:O_2 2:1.

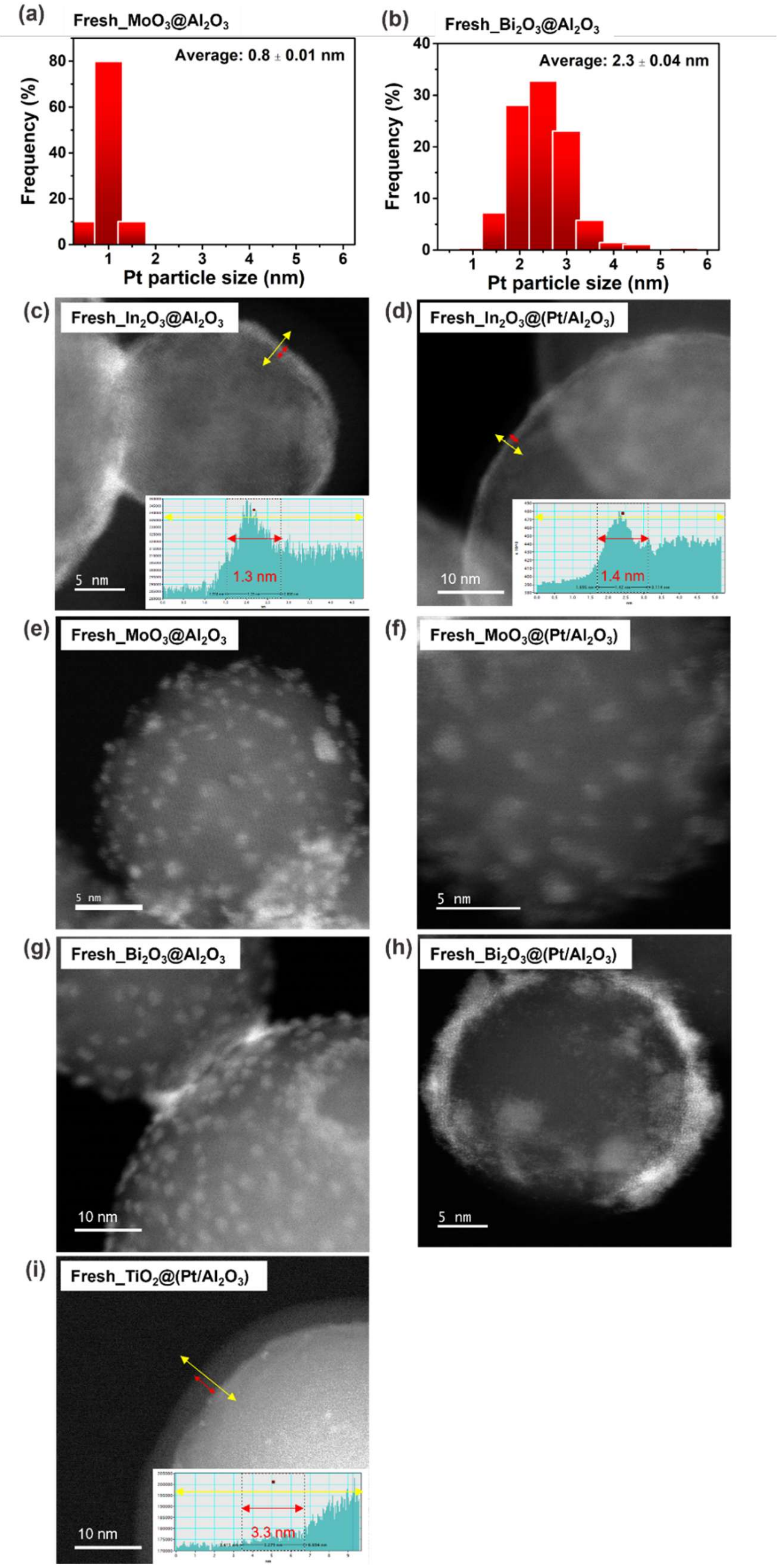

Figure S8. Particle size distributions of (a) MoO_3 and (b) Bi_2O_3 clusters over MoO_3@Al_2O_3 and Bi_2O_3@Al_2O_3 nanomaterials. (c-i) Magnified HAADF-STEM images of freshly-prepared, overcoated nanomaterials.

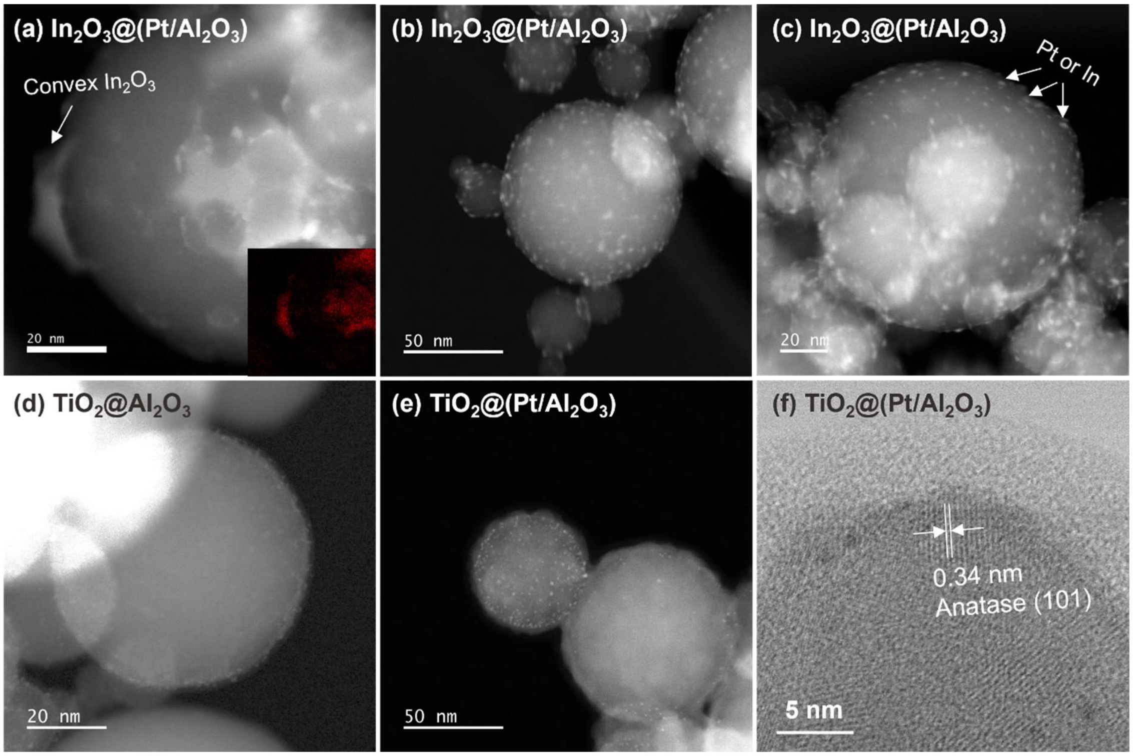

Figure S9. STEM/HRTEM images of (a–c) the In_2O_3@(Pt/Al_2O_3) and (d) TiO_2@Al_2O_3, and (e and f) TiO_2@(Pt/Al_2O_3) material after 450°C in 2:1 propane:O_2. **Figures S9a–c** show different types of restructured In_2O_3 nanostructures, indicating strong interaction with Pt nanoparticles.

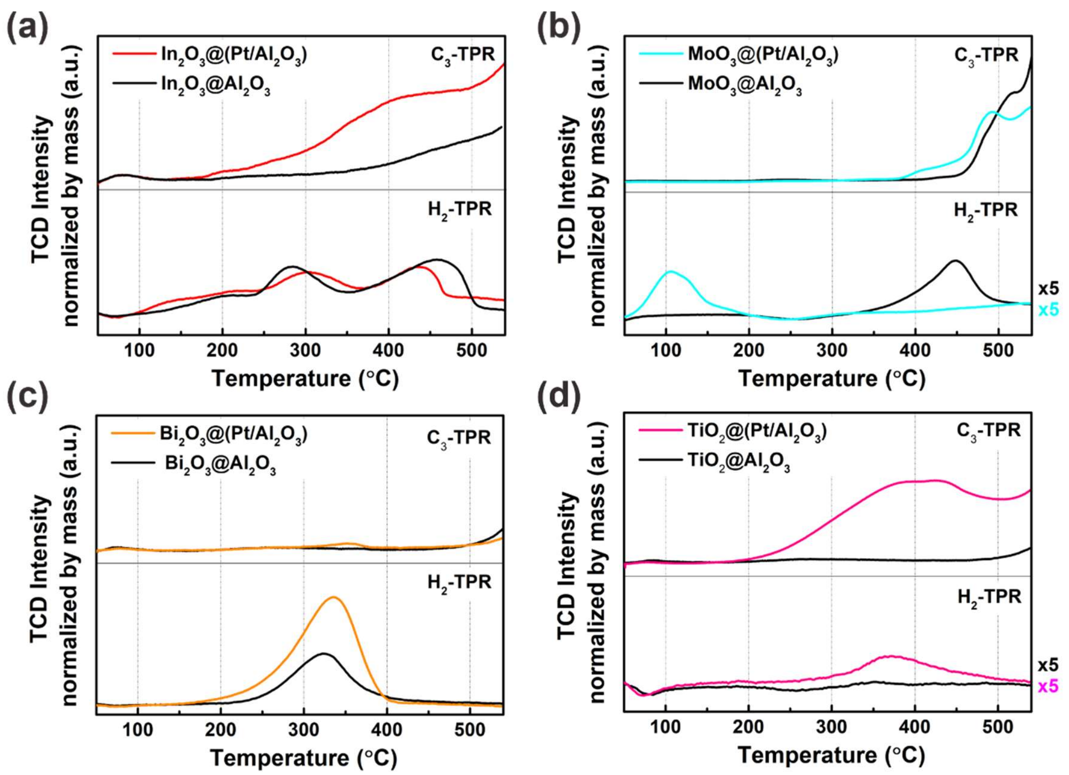

Figure S10. TCD signals for the H_2-TPR and propane (C_3)-TPR profiles of MOx@Al_2O_3 and MOx@(Pt/Al_2O_3) (MOx = In_2O_3 (a), MoO_3 (b), Bi_2O_3 (c), and TiO_2 (d)) after pretreatment at 200°C under He for 1 h. The y-axis is normalized to the total mass of the material.

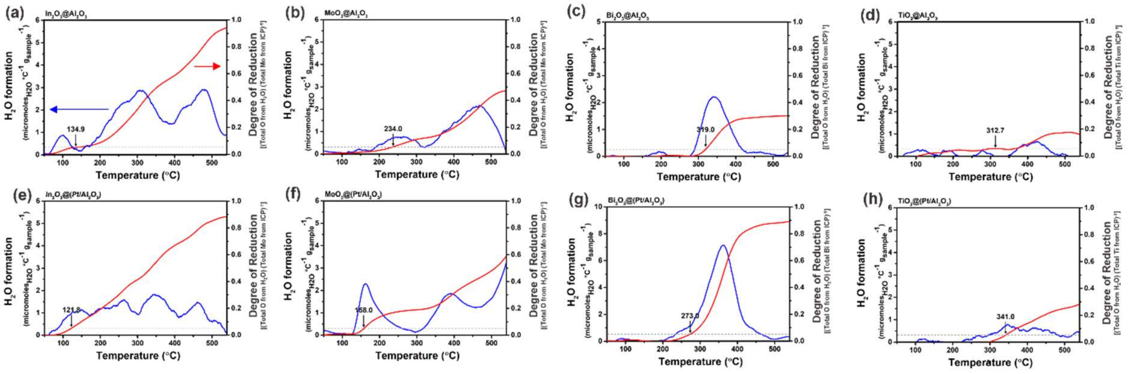

Figure S11. Mass spectrometer traces for H_2O (m/z = 18) during H_2-TPR of ALD overcoated nanomaterials and the corresponding degree of reduction (DOR) as a function of temperature. (a) In_2O_3@Al_2O_3, (b) MoO_3@Al_2O_3, (c) Bi_2O_3@Al_2O_3, (d) TiO_2@Al_2O_3, (e) In_2O_3@(Pt/Al_2O_3) (f) MoO_3@(Pt/Al_2O_3), (g) Bi_2O_3@(Pt/Al_2O_3), and (h) TiO_2@(Pt/Al_2O_3).

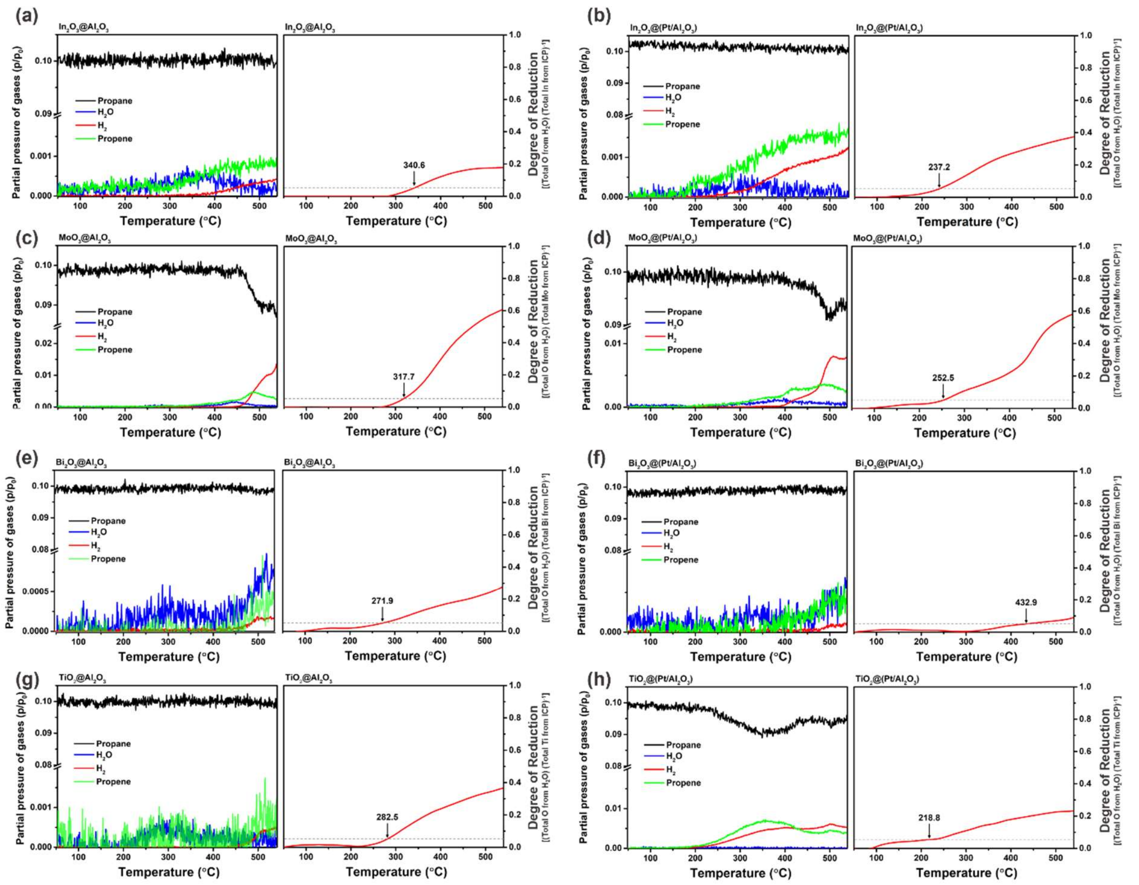

Figure S12. Mass spectrometer traces for H_2O (m/z = 18), H_2 (m/z = 2), C_3H_6 (m/z = 42) and C_3H_8 (m/z = 44) during propane-TPR of ALD overcoated nanomaterials. The partial pressure of each gas component is shown in the left panel and the degree of reduction, obtained by using the partial pressure of H_2O, is shown in the right panel. (a) In_2O_3@Al_2O_3, (b) In_2O_3@(Pt/Al_2O_3), (c) MoO_3@Al_2O_3, (d) MoO_3@(Pt/Al_2O_3), (e) Bi_2O_3@Al_2O_3, (f) Bi_2O_3@(Pt/Al_2O_3), (g) TiO_2@Al_2O_3, and (h) TiO_2@(Pt/Al_2O_3).

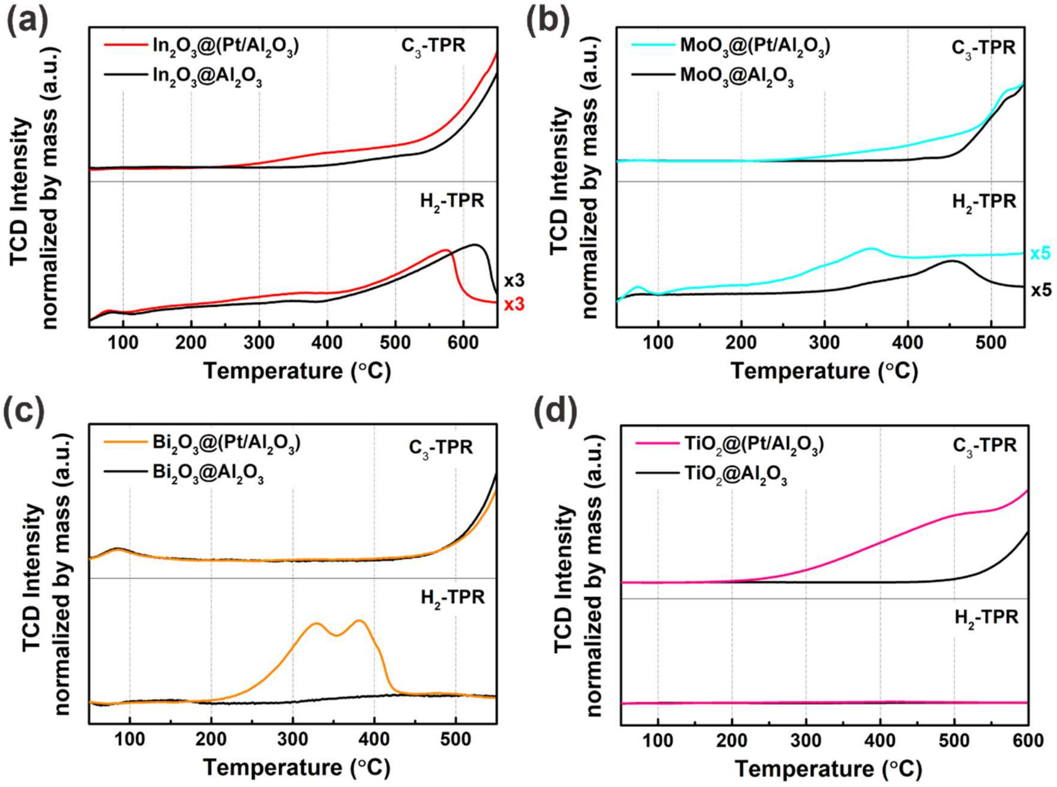

Figure S13. TCD signals for H_2-TPR and propane (C_3)-TPR profiles of MOx@Al_2O_3 and MOx@(Pt/Al_2O_3) (MOx = In_2O_3 (a), MoO_3 (b), Bi_2O_3 (c), and TiO_2 (d)) after pretreatment at elevated 450°C under He for 1 h. The y-axis is normalized to the total mass of the material.

Acknowledgments

The authors gratefully acknowledge support from the Institute for Catalysis in Energy Processes, funded by DOE, Office of Science, Office of Basic Energy Sciences (DE-FG02-03ER15457). This work was partially funded by the Trienens Institute for Sustainability and Energy at Northwestern University. This work made use of the Reactor Engineering and Catalyst Testing (REACT) core facility of the Center for Catalysis and Surface Science at Northwestern University. This work made use of the NUFAB facility of Northwestern University's NUANCE Center, which has received support from the SHyNE Resource (NSF ECCS-2025633), the IIN, and Northwestern's MRSEC program (NSF DMR-1720139). K.K. acknowledges support from the Air Force Office of Scientific Research (AFOSR) with the grant number of AFOSR FA9550-22-1-0300. This work made use of the EPIC and NUFAB facility of Northwestern University's NU*ANCE* Center, which has received support from the SHyNE Resource (NSF ECCS-2025633), the IIN, and Northwestern's MRSEC program (NSF DMR- 2308691). Elemental analysis was performed at the Northwestern University Quantitative Bio-element Imaging Center. This work made use of the IMSERC X-RAY facility at Northwestern University, which has received support from the Soft and Hybrid Nanotechnology Experimental (SHyNE) Resource (NSF ECCS-2025633), and Northwestern University.

References

1. Yan, H. *et al.* Tandem In_2O_3-Pt/Al_2O_3 catalyst for coupling of propane dehydrogenation to selective H $_2$ combustion. *Science* **371**, 1257–1260 (2021).
2. Libera, J. A., Hryn, J. N. & Elam, J. W. Indium Oxide Atomic Layer Deposition Facilitated by the Synergy between Oxygen and Water. *Chem. Mater.* **23**, 2150–2158 (2011).
3. Wang, C. *et al.* Influence of plasma power on deposition mechanism and structural properties of MoO *x* thin films by plasma enhanced atomic layer deposition. *J. Vac. Sci. Technol. Vac. Surf. Films* **39**, 032415 (2021).
4. Qiao, Q., Li, Y. W., Zhang, J. Z., Hu, Z. G. & Chu, J. H. Experimental investigations of the bismuth oxide film grown by atomic layer deposition using triphenyl bismuth. *Thin Solid Films* **622**, 65–70 (2017).
5. Hatanpää, T., Vehkamäki, M., Ritala, M. & Leskelä, M. Study of bismuth alkoxides as possible precursors for ALD. *Dalton Trans.* **39**, 3219 (2010).
6. Shen, Y. D. *et al.* Growth of Bi $_2$ O $_3$ Ultrathin Films by Atomic Layer Deposition. *J. Phys. Chem. C* **116**, 3449–3456 (2012).
7. Wang, C. *et al.* Precisely Applying TiO $_2$ Overcoat on Supported Au Catalysts Using Atomic Layer Deposition for Understanding the Reaction Mechanism and Improved Activity in CO Oxidation. *J. Phys. Chem. C* **120**, 478–486 (2016).
8. Lee, J., Jang, E. J., Oh, D. G., Szanyi, J. & Kwak, J. H. Morphology and size of Pt on Al2O3: The role of specific metal-support interactions between Pt and Al_2O_3. *J. Catal.* **385**, 204–212 (2020).
9. Zhang, X. *et al.* Radical Chemistry and Reaction Mechanisms of Propane Oxidative Dehydrogenation over Hexagonal Boron Nitride Catalysts. *Angew. Chem. Int. Ed.* **59**, 8042–8046 (2020).
10. Motakef-Kazemi, N. & Yaqoubi, M. Green synthesis and characterization of bismuth oxide nanoparticle using mentha pulegium extract. *Iran. J. Pharm. Res.* (2020) doi:10.22037/ijpr.2019.15578.13190.
11. Mishra, M. *et al.* Switchable surface activity of $Bi_2Al_4O_9$ nano particles: A contemporary approach in heterocyclic synthesis. *J. Nanoparticle Res.* **25**, 49 (2023).
12. Son, J. Y., Park, C. S. & Shin, Y.-H. Epitaxial $BiAlO_3$ thin film as a lead-free ferroelectric material. *Appl. Phys. Lett.* **92**, 222911 (2008).
13. Topham, B. J., Kumar, M. & Soos, Z. G. Ionization potentials of crystalline organic thin films: Position dependence due to molecular shape and charge redistribution. *Chem. Phys. Lett.* **493**, 251–254 (2010).
14. Zhou, Y. *et al.* Monolayered Bi_2WO_6 nanosheets mimicking heterojunction interface with open surfaces for photocatalysis. *Nat. Commun.* **6**, 8340 (2015).
15. Shido, T., Okita, G., Asakura, K. & Iwasawa, Y. Preparation, Characterization, and Catalytic Performance of Bismuth−Aluminum Binary-Oxide Layers and Clusters on an Al_2O_3 Surface. *J. Phys. Chem. B* **104**, 12263–12268 (2000).
16. Wei, H. *et al.* Molybdenum Carbide Nanoparticles Coated into the Graphene Wrapping N-Doped Porous Carbon Microspheres for Highly Efficient Electrocatalytic Hydrogen Evolution Both in Acidic and Alkaline Media. *Adv. Sci.* **5**, 1700733 (2018).
17. Shen, C. *et al.* Highly Active Ir/In_2O_3 Catalysts for Selective Hydrogenation of CO_2 to Methanol: Experimental and Theoretical Studies. *ACS Catal.* **11**, 4036–4046 (2021).
18. Dinesh Kumar, D. *et al.* Probing the Impact of Tribolayers on Enhanced Wear Resistance Behavior of Carbon-Rich Molybdenum-Based Coatings. *ACS Appl. Mater.*

Interfaces **14**, 26148–26161 (2022).
19. Meng, F., Xiao, L. & Sun, Z. Thermo-induced hydrophilicity of nano-TiO_2 thin films prepared by RF magnetron sputtering. *J. Alloys Compd.* **485**, 848–852 (2009).
20. Fan, C. *et al.* Black Hydroxylated Titanium Dioxide Prepared via Ultrasonication with Enhanced Photocatalytic Activity. *Sci. Rep.* **5**, 11712 (2015).
21. Enger, B. C., Lødeng, R., Walmsley, J. & Holmen, A. Inactive aluminate spinels as precursors for design of CPO and reforming catalysts. *Appl. Catal. Gen.* **383**, 119–127 (2010).
22. Liu, Y. *et al.* One-step synthesis of defected $Bi_2Al_4O_9$/β-Bi_2O_3 heterojunctions for photocatalytic reduction of CO_2 to CO. *Green Energy Environ.* **6**, 244–252 (2021).
23. Fan, H., Wang, G. & Hu, L. Infrared, Raman and XPS spectroscopic studies of Bi2O3–B_2O_3–Ga_2O_3 glasses. *Solid State Sci.* **11**, 2065–2070 (2009).
24. Shimizugawa, Y., Sugimoto, N. & Hirao, K. X-ray absorption fine structure glasses containing Bi_2O_3 with third-order non-linearities. *J. Non-Cryst. Solids* **221**, 208–212 (1997).
